# Environmental Correlation Analysis for Genes Associated with Protection against Malaria

**DOI:** 10.1093/molbev/msw004

**Published:** 2016-01-06

**Authors:** Margaret J. Mackinnon, Carolyne Ndila, Sophie Uyoga, Alex Macharia, Robert W. Snow, Gavin Band, Anna Rautanen, Kirk A. Rockett, Dominic P. Kwiatkowski, Thomas N. Williams

**Affiliations:** ^1^Department of Epidemiology and Demography, KEMRI-Wellcome Trust Research Programme, Kilifi, Kenya; ^2^Department of Public Health Research, KEMRI-Wellcome Trust Research Programme, Nairobi, Kenya; ^3^Centre for Tropical Medicine, Nuffield Department of Clinical Medicine, University of Oxford, Oxford, United Kingdom; ^4^Wellcome Trust Centre for Human Genetics, Nuffield Department of Medicine, University of Oxford, Oxford, United Kingdom; ^5^The Wellcome Trust Sanger Institute, Cambridge, United Kingdom; ^6^Department of Medicine, Imperial College, London, United Kingdom; ^7^INDEPTH Network, Kanda, Accra, Ghana

**Keywords:** environmental correlation analysis, balancing selection, malaria, local adaptation

## Abstract

Genome-wide searches for loci involved in human resistance to malaria are currently being conducted on a large scale in Africa using case-control studies. Here, we explore the utility of an alternative approach—“environmental correlation analysis, ECA,” which tests for clines in allele frequencies across a gradient of an environmental selection pressure—to identify genes that have historically protected against death from malaria. We collected genotype data from 12,425 newborns on 57 candidate malaria resistance loci and 9,756 single nucleotide polymorphisms (SNPs) selected at random from across the genome, and examined their allele frequencies for geographic correlations with long-term malaria prevalence data based on 84,042 individuals living under different historical selection pressures from malaria in coastal Kenya. None of the 57 candidate SNPs showed significant (*P* < 0.05) correlations in allele frequency with local malaria transmission intensity after adjusting for population structure and multiple testing. In contrast, two of the random SNPs that had highly significant correlations (*P* < 0.01) were in genes previously linked to malaria resistance, namely, *CDH13*, encoding cadherin *13*, and *HS3ST3B1*, encoding heparan sulfate 3-O-sulfotransferase 3B1. Both proteins play a role in glycoprotein-mediated cell-cell adhesion which has been widely implicated in cerebral malaria, the most life-threatening form of this disease. Other top genes, including *CTNND2* which encodes δ-catenin, a molecular partner to cadherin, were significantly enriched in cadherin-mediated pathways affecting inflammation of the brain vascular endothelium. These results demonstrate the utility of ECA in the discovery of novel genes and pathways affecting infectious disease.

## Introduction

Although many genes affecting susceptibility to malaria have been reported in the literature (Kwiatkowski 2005), their validation in large, multisite, genome-wide phenotype–genotype association studies (GWAS) has been disappointing, producing only weak signals (Jallow et al. 2009; Timmann et al. 2012; MalariaGEN 2014) or inconsistent results across different studies (Atkinson et al. 2007; Cserti and Dzik 2007; Fry, Auburn, et al. 2008; Clark et al. 2009; Mangano et al. 2009; Teo et al. 2010; MalariaGEN 2014). For example, of the 57 single nucleotide polymorphism (SNP) loci representing 39 “candidate” genes selected for the first phase of the MalariaGEN Consortium large multipopulation case-control studies (MalariaGEN 2008), only five of these loci—the sickle cell-causing allele of the beta-hemoglobin gene (*HBB*), the “O” allele in the *ABO* gene that determines ABO blood group, both of which were well established as malaria protective prior to the advent of GWAS studies, *G6PD*, *CD40LG*, and *ATP2B4*—were confirmed (MalariaGEN 2014).

One explanation for the discrepancies between results from large, multipopulation GWAS, and single-site studies may lie in the profound degree of genetic diversity seen over very small distances in African populations (Tishkoff and Williams 2002) which, due to undetected population structure, coupled with variation in disease transmission, can generate both false positive and false negative results (Marchini et al. 2004). Another explanation is the lack of power of marker-based genome scans as a consequence of low levels of linkage disequilibrium in African genomes (Conrad et al. 2006; Jallow et al. 2009). Improved statistical methods such as genotype imputation, meta-analyses that allow for heterogeneous gene effects in different populations, and adjustment for population structure using information on thousands of genetic markers can alleviate these problems, though only partially (Band et al. 2013). Further improvements in methodology are required in order to find the many polymorphic genes affecting susceptibility to malaria that, apparently, still await discovery (Kwiatkowski 2005; Mackinnon et al. 2005).

An alternative approach to detecting disease-protective alleles using phenotype–genotype association methods such as case-control studies is to examine population patterns of allele frequencies in relation to an environmental variable using “environmental correlation analysis” (ECA). Stimulated by the recent advent of genome-wide technologies, this “landscape genomics” approach has been successfully pioneered in searches for new genes conferring environment-specific adaptation in humans, for example, to temperature, altitude, and diet (Novembre and Di Rienzo 2009; Coop et al. 2010; Hancock et al. 2010; Pritchard et al. 2010), and in a diverse range of plant (Eckert et al. 2010; Manel et al. 2010; Hancock et al. 2011) and animal species (Foll and Gaggiotti 2008; Nielsen et al. 2009). The principle behind ECA is that when there is differential positive selection pressure on an allele in spatially separated populations brought about by geographical variation in environmental conditions, and there is also negative selection on the allele due to a fitness cost, the allele will, through balancing selection, be maintained at intermediate population frequencies which correlate to the strength of selection by the environmental variable. This “fine tuning” form of adaptation through subtle frequency changes in alleles with modest protective effects contrasts with the “hard sweep” model of adaptation in which mutations with large beneficial effect always approach fixation, even if they carry a moderate fitness cost (Pritchard et al. 2010).

Given that human populations in Africa have been exposed to malaria under a range of transmission intensities for a very long time, and that many malaria-protective genes are only found at detectable frequencies in populations where malaria occurs and are therefore likely to carry a malaria-unrelated fitness cost, we reasoned that adaptation to malaria was more likely to follow the fine-tuning model of adaptation than the hard sweep model. We therefore wanted to test whether frequencies of malaria-protective alleles track malaria transmission intensity in an African population at equilibrium in its natural disease setting. While positive correlations between malaria transmission intensity and frequency of malaria resistance alleles have been demonstrated at a global scale for several hemoglobinopathy causing genes (Weatherall and Clegg 2001; Piel et al. 2010), an observation that led to Haldane’s famous “malaria-hypothesis” (Haldane 1949), there has been much debate on whether this principle holds at a local geographic scale (Allison 1954; Foy et al. 1954; Moore et al. 1954; Raper 1954; Brass et al. 1955; Siniscalco et al. 1966; Flint et al. 1986, 1993; Enevold et al. 2007). Counter-arguments are that confounding between the effects of migration, origin of the mutation, genetic admixture, nonindependence of population samples, and ecological suitability for malaria have generated spurious geographic relationships between malaria transmission intensity and allele frequencies (Flint et al. 1998). This problem is general to all geographic-genetic association studies and is not able to be overcome through statistics alone (Novembre and Di Rienzo 2009). However, ECA exploits these geographic-genetic correlations and thus, when combined with a recent statistical advance (Coop et al. 2010; Gunther and Coop 2013) that takes into account the neutral processes that generate allele frequency differences among populations, is well suited to disentangling adaptive from spurious clines in allele frequencies across an environmental gradient.

Here, we sought evidence for geographic correlations between malaria exposure and allele frequencies of 57 SNPs representing 39 candidate malaria-protective genes, and nearly 10,000 random SNP loci representing 3,010 protein-coding genes, in a geographically small but genetically and environmentally heterogeneous population living on the coast of Kenya. Our aim was to test whether, as for metabolic traits in humans driven by environment-related selection pressures (Novembre and Di Rienzo 2009; Coop et al. 2010; Hancock et al. 2010; Pritchard et al. 2010), the ECA approach can detect genes that confer resistance to infectious diseases. We further wished to determine whether it could do so on a small geographic scale in the genetically and environmentally heterogeneous populations which are typical of infectious disease study sites in Africa.

## Results

### Malaria Prevalence

**Fig. 1.**
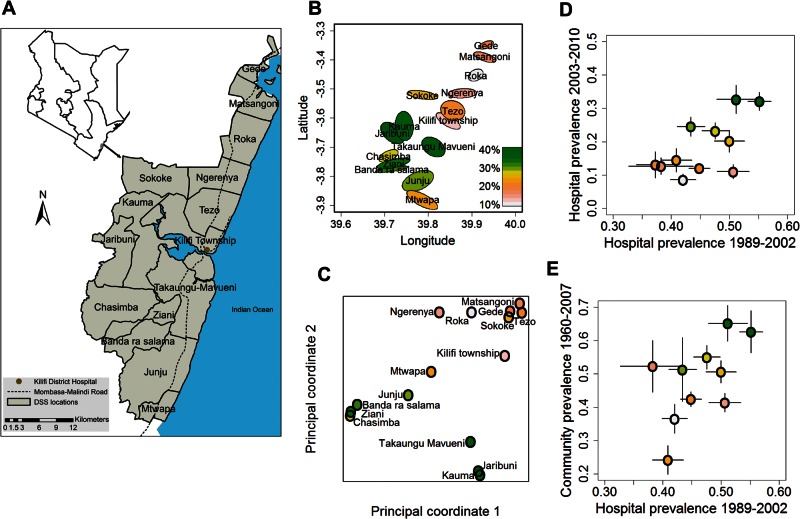
Geographic, genetic, and malaria prevalence maps of the study population. (*A*) Geographic map of study area showing boundaries of the 15 subpopulations. (*B*) Mean and 95% CI (center and boundary of colored ellipses) of geographic coordinates of residents of all children in the genotyped birth cohort. (*C*) Genetic map of the subpopulations based on multidimensional scaling analysis of data from 9,756 random SNP loci in the genotyped controls from the case-control cohort, rescaled to that of the geographic map in (*B*). (*D*) Malaria prevalence by subpopulation among hospital admissions from 2003 to 2010 (*y* axis) versus that in 1989 to 2002 (*x* axis). Symbols show year-adjusted means: black lines either side indicate 95% CI. (*E*) Malaria prevalence by subpopulation from community surveys from 1960 to 2007 (*y* axis) against hospital malaria prevalence in 1989 to 2002 (*x* axis). Regression analysis of these preadjusted means revealed consistent ranking in subpopulation malaria prevalence when assessed by community surveys versus hospital data in 1989–2002 (*P* = 0.08) and in 2003–2010 (*P* = 0.006), and when measured in two different time periods, that is, hospital data from 1989 to 2002 versus 2003 to 2010 (*P* = 0.03). (See supplementary fig. S1, Supplementary Material online for further detail.) Throughout, colors of points indicate mean malaria prevalence among hospital admissions in 2003–2010 according to the legend in (*B*). The fewer points in (*D*) and (*E*) than in (*B*) and (*C*) are due to the fact that some subpopulations were split in 2003, as described in the legend to supplementary figure S2, Supplementary Material online.

Long-term malaria prevalence, based on an average of 3,988 records per subpopulation collected over a period of 50 years (table 1), differed substantially between subpopulations (fig. 1). These differences were stable through time, with a high average correlation between years (0.54), despite very large changes in average malaria prevalence during this period (fig. 1*D* and supplementary fig. S1, Supplementary Material online). Subpopulation differences were also robust to the method of data collection, that is, from hospital admissions versus from community surveys (fig. 1*E*). Assuming that these geographic differences in malaria prevalence were also present in previous generations of our study population, these results establish the first requirement for the existence of malaria-related clines in frequencies of protective alleles, namely, stable, long-term geographical heterogeneity in selection pressure by malaria.

**Table 1.** **Data Sets Used for ECA**.

Genotype Data			Malaria Prevalence Data		
Status	**No. of Individuals**		**Data Source**	**No. of Subpopulations**	**No. of Records**
	**Random Loci**	**Candidate Loci**^a^			
All cases and controls	5,214	12,425 (3,868)	All	11	82,042
Controls only	2,927	10,597 (2,896)	Hospital admissions 1989–2002	11	42,429
Malaria cases only	1,063	1,832 (974)	Hospital admissions 2003–2010	11	19,282
Bacteremia cases only	1,133	—^b^	Hospital admissions 2003–2010	15	19,282
			Hospital admissions 2003–2010	38	19,282
			Community surveys 1960–2007	10	20,331

^a^Number of overlapping individuals, that is, with genotype data for both random and candidate loci, are shown in parentheses.

^b^Genotype data on candidate genes were not available for bacteremia cases.

### Population Structure

The second requirement for the maintenance of clines—geographic heterogeneity in host genetics—was revealed by hierarchical cluster analysis of between-subpopulation correlations based on 9,756 random SNPs genotyped in control individuals from two case-control studies (*N* = 2,927) (supplementary fig. S2, Supplementary Material online). Three broad genetic clusters were found and these broadly coincided with the three main ethnic groups in the study population—Chonyi, Kauma, and Giriama—that reside predominantly in the south, middle, and north of the study area, respectively. Genetic distances between subpopulations, estimated by multidimensional scaling based on genotype data as above, broadly corresponded to the degree of geographic separation between subpopulations along a north-east to south-west transect (fig. 1*C*). Malaria generally increased across this transect (fig. 1*B*) thus generating some confounding between genetics, geography, and malaria transmission. This was partly mitigated by the presence of the genetic outlier populations of Jaribuni and Kauma in the center of the study area where malaria transmission intensities were highest (fig. 1), thus providing leverage for distinguishing between the null versus alternative hypotheses under test here, namely, that clines arose as a consequence of historical migration and incomplete admixture across the study area versus clines were generated through differential selection pressure by malaria.

Thus overall, the study population displayed genetic structuring that partially aligned to geographic distance. Subpopulations were not completely genetically isolated, however, evident from the generally low correlations in allele frequencies among subpopulations (median absolute value of 0.23) and substantial blending of ethnic group composition across subpopulations (supplementary fig. S2, Supplementary Material online). Thus the second condition for maintenance of adaptive clines by selection against the eroding effects of gene flow—incomplete panmixia—was also met.

### Malaria-Related Clines in Allele Frequencies of Candidate Malaria Resistance Genes

**Fig. 2.**
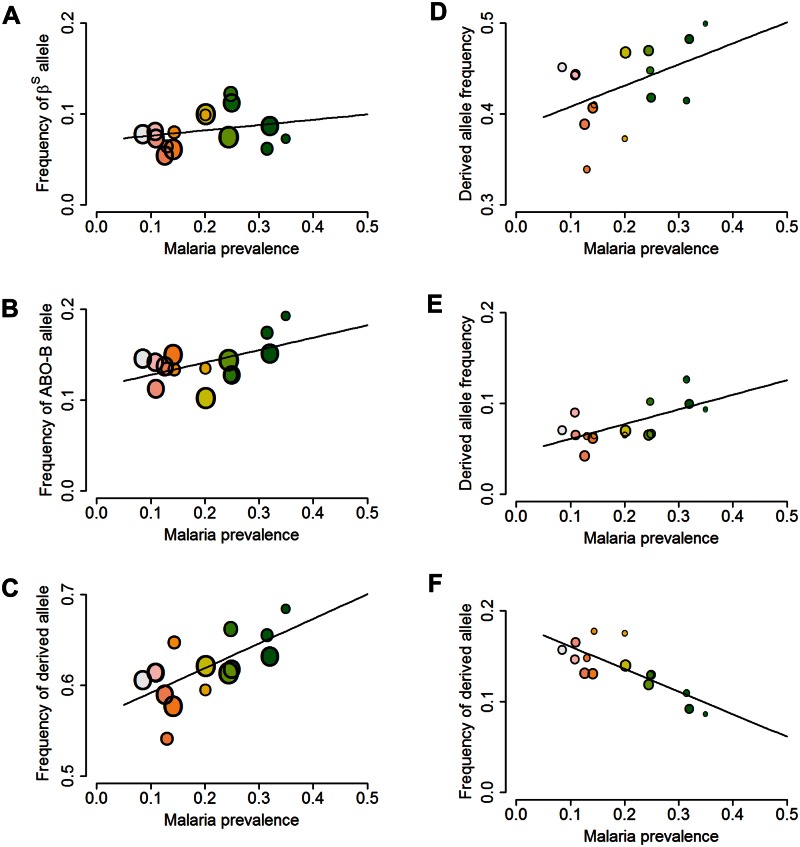
Malaria-related clines in allele frequencies in three candidate and three random loci. Relationship between malaria prevalence and allele frequency by subpopulation of (*A*) the sickle cell mutation (β^S^ allele), (*B*) the *ABO* rs8176746 allele that codes for the B versus A phenotype in the ABO blood group system, (*C*) the derived allele (T) in SNP rs2227478 in the *IL22* gene locus, (*D*) the derived allele in SNP rs8048962 in the *CDH13* gene, (*E*) the derived allele (C) in SNP rs4791574 in the *HS3ST3B1* gene, and (*F*) the derived allele (T) in SNP rs13358276 in the *CTTND2* gene. The solid lines show the fit of these relationships estimated by binomial logistic regression of individual allele data not accounting for population structure. Colors reflect malaria prevalence as in figure 1*B* and size of points scale with the square root of the number of genotypes. Results are based on all available genotype data from control individuals for the locus in question and hospital malaria prevalence data from 2003 to 2010 split into 15 subpopulations in order to correspond with data in figure 1.

Allele frequencies differed significantly between subpopulations for 23 of the 57 candidate loci (*P* < 0.05 by chi-squared test from logistic regression analyses fitting subpopulation as a fixed effect). For 15 of these loci, this variation between subpopulations was significantly related to malaria prevalence (*P* < 0.05 from logistic regression analyses fitting malaria prevalence as a linear covariate), that is, showed environmental correlations. Figure 2A–C shows examples of malaria-related frequency clines for the two of the 57 candidate genes that have consistently show genome-wide significance in case control studies (Jallow et al. 2009; Band et al. 2013; MalariaGEN 2014), namely, *HBB* and *ABO*, and for the SNP in the *IL22* gene that ranked highest for malaria-related clines here.

**Fig. 3.**
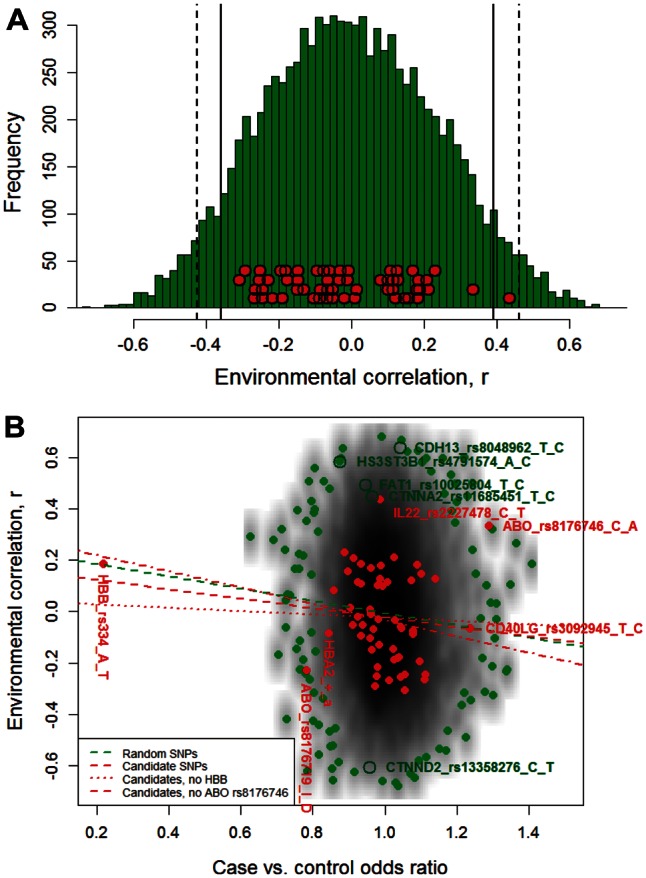
Environmental correlations for 57 malaria resistance candidate SNPs and 9,756 random SNPs and their relationship with case-control estimates of malaria-protective effects. (*A*) The green histogram shows the density distribution of the environmental correlation values, *r*, for 9,756 random SNP loci. Red circles with black outlines indicate values of *r* for the 57 candidate loci SNPs. Vertical solid and dashed black lines indicate the 5% and 2.5% tails of the distribution for the random SNPs, respectively. Values are based on all malaria prevalence data and genotype data from all case and control individuals using the same number of observations for candidate and random loci to ensure comparability. (*B*) Environmental correlations (*y* axis) were regressed on odds ratios of severe malaria in cases versus controls (*x* axis) obtained by logistic regression analysis of a subset of the data used for ECA. Odds ratios reflect the relative probabilities of carrying the derived allele in cases versus controls (*x* axis), and thus decrease as the allele’s protective effect against malaria increases. Spearman correlations between variables on the *x* and *y* axes were −0.23 (*P* = 0.09, 55 df) for candidate gene SNPs, and −0.08 (*P* < 0.001, 9,406 df) for random SNPs. Best-fit linear regression lines are shown with and without two candidate locus outliers (SNP rs334 at the *HBB* locus and SNP rs8176746 at the *ABO* locus, see legend). Depth of gray shading reflects the local regional density of observations for the random SNPs calculated using the smoothScatter function in the R “graphics” package (R Core Team 2014). Closed green symbols indicate the 1% of random SNPs with the most extreme values based on their having lowest regional densities. Closed red symbols indicate values for candidate gene SNPs. Candidate SNP outliers for protective efficacy are annotated with red text. Outliers for environmental correlations in random SNPs which are discussed in the main text are shown with open green symbols and annotated with green text. Global test statistics on estimates of protective effects indicate that the candidate loci had more extreme odds ratios than the random loci (*P* < 0.001, *P* = 0.005 excluding rs334 in the *HBB* gene). The distribution of environmental correlations for candidate loci was similar to that for random SNP loci (supplementary fig. S4*A*, Supplementary Material online).

However, the above logistic regression analyses above do not allow for the fact that populations differ in allele frequencies for reasons unrelated to selection and measurement error such as genetic drift, migration, and other neutral population genetics processes. When background variation between populations was taken into account by fitting an appropriate population genetics model using the Bayenv method (Coop et al. 2010; Gunther and Coop 2013), none of the 57 candidate loci reached the two-tailed *P* < 0.05 significance, and only one (SNP rs2227478 in the *IL22* gene) reached the *P* < 0.10 level (supplementary table S2, Supplementary Material online). This was true whether correlations were computed for all available genotype data (*N* = 10,597, the “full data set”), in which case asymptotic significance tests for Pearson correlations, which are conservative since they assume zero error of measurement in subpopulation frequencies, were applied, or whether correlations were computed from the same number of genotyped individuals as random SNP loci (*N* = 2,927, the “reduced data set”), in which case empirical significance tests based on the distribution of correlations among random SNPs were used (fig. 3A). After SNP rs2227478 in the *IL22* gene, which had environmental correlations of *r* = 0.59 and *r* = 0.43 for the full and reduced genotype data sets, respectively, with corresponding asymptotic and empirical *P* values of 0.08 and 0.06, the next highest ranking candidate gene SNPs were rs8176746 that codes for the B allele in the *ABO* locus (*r* = 0.48, *P* = 0.14) and rs2535611 in the *ADORA2B* gene (*r* = −0.35, *P* = 0.29) (values based on the full genotype data set; supplementary table S2 and fig. S4*A*, Supplementary Material online).

When a test for general population differentiation, that is, irrespective of malaria, was applied using the X^T^X statistic described by Gunther and Coop (2013), significant (*P* < 0.05) signals were found for SNPs rs1128127 in the *DERL3* gene and rs84833095 in the *TLR1* gene, and marginally significant signals (*P* < 0.10) were found for SNPs rs2706384 in the *IRF1* gene and rs1803632 in the *GBP7* gene (supplementary fig. S4*B*, Supplementary Material online).

Consistent with the few candidate loci showing individual significance for *r* and *X^T^X* was the general lack of significance of global tests of whether the 57 (or top six) candidate loci, as a set, showed more extreme correlations than random sets of 57 (or top six) of the 9,756 random loci (supplementary fig. S4, Supplementary Material online). For some analyses, however, such as when the population was divided into 38 instead of 15 or 11 subpopulations, global tests indicated that candidate SNPs had, as a set, values of *r* significantly lower in magnitude and values of *X^T^X* higher in magnitude than random SNPs (supplementary fig. S4, Supplementary Material online).

### Environmental Correlations in Random SNP Loci

Among the top 1% (*n* = 98) random SNP loci for ECA signals were two genes that have previously shown strong genetic associations with malaria (supplementary table S2, Supplementary Material online). The first was in *HS3ST3B1* (SNP rs4791574, *r* = 0.58, *P* = 0.007, fig. 2E) which encodes heparan sulfate (glucosamine) 3-O-sulfotransferase 3B1, an enzyme that modifies the sulfation patterns of the glycoprotein heparan sulfate. This SNP falls within an intron of *HS3ST3B1*. In a previous study in West Africa, 20 SNPs within the exons and 3′- and 5′-untranslated regions of this gene and its paralogous neighboring gene *HS3ST3A1* were strongly associated with malaria parasitemia (Atkinson et al. 2012). Contrary to expectations from the direction of the environmental correlation, there was a strong deficit in frequency of the derived (malaria-favored) allele relative to the global population (0.07 vs. 0.36).

A second gene among the top 1% random loci that has been previously implicated as malaria protective (Band et al. 2013) was *CDH13* (rs8048962, *r* = 0.64, *P* = 0.0013, fig. 2D) which encodes cadherin 13, a member of a large family of proteins that mediate cell-cell adhesion as well as extracellular signaling, most known for their role in neural cells but which also operate in the vasculature and other tissues. A further SNP in this gene showed a strong environmental correlation with malaria (rs4782731, *r* = 0.45, *P* = 0.055). This result confirms the finding from the MalariaGEN Consortium’s large multipopulation genome-wide case-control study for severe malaria in Africa in which *CDH13* was one of just 18 genes, excluding the candidate genes, which survived tests of genome-wide significance and replicability across populations (Band et al. 2013). Significant population differentiation in other SNPs in this gene has been reported in south-east Asian populations (Liu, Yunus, et al. 2015).

If it is assumed that there are 20 among the approximately 20,000 genes in the genome (∼0.1%) that strongly protect against malaria, given that our random set of 9,756 SNPs included 3,010 characterized protein-coding genes, it is expected that three of these malaria protective genes would be included in our survey. The probability by chance that exactly one of these three would be among the 42 protein-coding genes in the top 1% of SNPs (supplementary table S2, Supplementary Material online) is *P* = 0.04 by hypergeometric test: the probability that two or more were among these top 42 is *P* = 0.0006. Supporting these assumptions is the fact that our survey of random loci included three (*ZNF804A*, *CDH13*, *SIRT3*) of 18 novel malaria resistance genes recently uncovered by GWAS (Timmann et al. 2012; Band et al. 2013; MalariaGEN 2014; MalariaGEN et al. 2015) (*ATP2B4*, *MYOT*, *C10ord57*, *C11orf40*, *STIM1*, *MARVELD3* [Timmann et al. 2012], the linked genes *ODF3-BET1-RIC8A-SIRT3* and the linked genes *SMARCA5-FREM3-GYPE-GYPB-GYPA*, in addition to the three above).

Tests for functional enrichment among the top 10% of SNPs, which represented 382 protein-coding genes, revealed a significant excess of genes in the cadherin-catenin mediated cell-cell adhesion pathway to which *CDH13* belongs (*P* = 0.005, supplementary table S3, Supplementary Material online), as well as in the related pathways of G-protein coupled signaling that cadherins mediate in response to extra cellular signals from cytokines, chemokines, pathogens, and mechanical forces, including the rigidifying and inflammatory response of the vascular endothelium to shear stress and stretching (Birukov 2009); the metalloproteases that degrade extracellular matrix proteins, implicated in inflammation-related damage to the blood brain barrier (Bruschi and Pinto 2013); and O-linked glycosylation of proteins in the extracellular matrix that are involved in cell-cell adhesion. These pathways are of particular relevance because of the known role of inflammation and damage to permeability of the brain vascular endothelium in cerebral malaria (supplementary table S3, Supplementary Material online).

Individual genes of interest among the top 10% random loci were: *CDH5* (*r* = −0.44, *P* = 0.06) which encodes the cadherin most abundantly expressed in the vascular endothelium (hence also known as VE-cadherin) which plays a key role in vascular permeability and leakage (Gavard 2014); four SNPs within three protocadherin-encoding genes (*FAT1*, *PCDH9*, *PCDH15*); two SNPs in genes coding for catenins, the proteins that link cadherins to the cell cytoskeleton (*CTNND2*, *P* = 0.003, fig. 2F) which codes for δ-catenin that has been shown to mediate inflammation-related pathology of the vascular endothelium in the brain (DeBusk et al. 2010), and *CTTNNA2, P* = 0.05); *MAGI2* (*P* = .009) a homologue of *MAGI1* which encodes a linker molecule in the cadherin-catenin complex in the vascular endothelium (Wallez and Huber 2008) and which a variety of evidence suggests interacts with CTNND2 (Schmitt et al. 2014); *FN1* (*P* = 0.002) which encodes the plasma protein fibronectin that, like cadherins, binds extracellular matrix proteins, including heparan sulfate, and helps promote repair of the vascular endothelium after damage; three genes that encode regulators of G-protein signaling that is typically coupled to cadherin activity (*RGS5*, *RGS6* (*P = *0.01), *RGS7*), one of which (*RGS5*) has been shown to be involved in endothelial apoptosis (Jin et al. 2009); two genes encoding protein kinases of type C (*PRKCE*, *PRKCH*) that mediate signal transduction via G-coupled protein receptors, both associated with cerebral ischemia (Perez-Pinzon et al. 2005; Kubo et al. 2007) and one (*PRKCE*) of which is implicated in protection against the Kenya-endemic Rift Valley Fever virus (Filone et al. 2010); *ADGRL2* (*P* = 0.009) that encodes a G-protein coupled receptor involved in adhesion and signal transduction in immune regulation (Li et al. 2015) and which is closely related to *ADGRL1* which is highly expressed in the brain and closely related to one of the malaria resistance candidate genes studied here (*EMR1*); *ANGPT4* which encodes a receptor for tyrosine-protein kinases that regulate endothelial cells in the vasculature in response to inflammation and structural damage; *OPMCL* (two SNPs, *P* = 0.009 and *P* = 0.01), encoding a receptor found at high levels in the brain and which regulates tyrosine kinases (Wu and Sood 2012); *NRP1* (*P* = 0.005), which codes for neuropilin 1, a protein that acts as a receptor for vascular endothelial growth factor in association with a tyrosine kinase coreceptor and plays a role in angiogenesis; *NRXN3* (*P* = 0.008), a gene encoding a receptor and signaling molecule that is highly expressed in the brain; a gene encoding diacylglycerol lipase (*DAGLA*) that hydrolyses diacylglycerol, an activator of protein kinase C; four genes for cytokines or their regulators involved in the immune response to microbial infection and inflammatory responses (*IRF2*, coding for a transcription factor that regulates interferon-γ; *IL12RA-AS1*, an antisense RNA that is expected to control levels of interleukin 12, a cytokine that acts on T and natural killer cells and has been genetically associated with malaria (Zhang et al. 2010); *IL2RA*, which encodes a receptor on lymphocytes that responds to the key cytokine in defense against microbes, IL2; and *IL34* which encodes a cytokine that is abundant in the spleen, a site of immune defence against malaria parasites); a gene encoding a component of the red cell cytoskeleton, ankyrin 3 (*ANK3*) and casein kinase 2A1 (*CSNK2A1*), both which help anchor the *Plasmodium falciparum* protein, PfEMP1, (Hora et al. 2009; Weng et al. 2014) to red cell membrane where it mediates cytoadhesion to uninfected red cells (Rowe et al. 1995) and other host cells (Magowan et al. 1988), including the vascular endothelium (Ockenhouse et al. 1992), a process which is thought to lead to cerebral malaria; two genes coding for myosin (*MYO16*, *MYO5C*); and five genes encoding collagens, one of which (*COL4A2*) has been associated with cerebral small vessel hemorrhage (Rannikmae et al. 2015).

Of relevance to the second confirmed malaria-associated gene among the top 1% of random SNPs (*HS3STSB1*), is the significant enrichment for the O-linked glycosylation pathway among the top 10% of SNPs (supplementary table S3, Supplementary Material online). One gene among the top 1% (*POMGNT1, r* = 0.62*, P* = 0.002) and four others in the top 10% (*GALNT2*, *GALNT5*, *GALNT10*, *GALNT13*) code for enzymes that mediate the same type of O-linked glycosylation (addition of N-acetyl-galactose amine) that converts the H antigen in the ABO blood group system to the A antigen instead of the B antigen which is determined by the addition of galactose. This glycosylation results in higher susceptibility to malaria compared with the O blood group for which neither of these glycosylations occur, and differential susceptibility of the A versus, B blood group (supplementary table S2, Supplementary Material online).

Other genes of interest in the top 1% that are not obviously related to the enriched pathways included those encoding transcriptional regulators (*SRPK2*, *P* < 0.00001, and *RBFOX1* (two SNPs, *P* = 0.0003 and *P* = 0.002) that both control alternative splicing, and *SSBP3* (*P* = 0.009) which binds DNA); *BICD1* (*P* = 0.006), which regulates Golgi-ER transport by recruiting dynein and dynactin; and *DNAH14* (*P* = 0.003), which encodes an axonemal dynein.

Further details of genes among the top 10% found in enriched pathways and their potential relevance to severe malaria are given in supplementary table S3, Supplementary Material online.

### Correspondence with Results from a Case-Control Study

Environmental correlations showed a significant positive relationship with malaria-protective effects directly estimated from the malaria case-control study nested within this study population (fig. 3B, *P* < 0.001 by Spearman rank correlation tests). Thus in addition to detecting novel loci (see above), ECA can be used to validate results from case-control studies. A notable exception to this general correspondence was SNP rs8176746 in the *ABO* gene which codes for the B blood group allele which by ECA is predicted to be protective against malaria but in the case-control study analyzed here, and in other case-control studies, when in combination with the A allele in AB heterozygotes, appears to confer susceptibility (supplementary table S2, Supplementary Material online).

Although obtained from the same population, the correspondence between estimates of *r* and the direct estimate of malaria-protective effects from the case-control study cannot be attributed to covariance introduced by the estimation procedure. This is because *r* and the malaria-protective effect were estimated independently using genotypes from different sets of individuals (on control individuals only in the case of *r*), as well as by independent statistical methods. The correspondence is also unlikely to be attributable to the negative correlation between slope and intercept that occurs when there is high sampling error due to small sample size, or to extreme allele frequencies, since these were not features of this study (supplementary text S1, Supplementary Material online).

### Replicability, Power, and Bias

Environmental correlation estimates for candidate loci were generally consistent in ranking across different genotype data sets (i.e., those based on control individuals only, malaria cases only, or cases and controls combined), and robust to sources of malaria prevalence data (hospital data vs. community surveys in different time periods, supplementary fig. S4*A*, Supplementary Material online). However, values of *r* were very sensitive to the number of genotyped individuals. Data simulation showed that both the magnitude and accuracy of individual correlation estimates scaled approximately linearly with the number of genotyped individuals (*N*) below *N* approximately 20,000 genotypes (i.e., double the size of the experiment reported here) and asymptotically beyond that (supplementary text S2, Supplementary Material online). This strong dependence of *r* on *N* has several important implications for the power and implementation of ECA. First, it requires equal numbers of genotyped individuals for each locus in order for correlations to be comparable across loci. This, in turn, means that efficiency of information usage is maximized when genotyping effort is evenly spread across loci. Second, it suggests that increasing *N* beyond the approximately 1,000 individuals genotyped per subpopulation used in this study is likely to yield considerable gains in power in situations where between-subpopulation variation in allele frequencies is low, as was the case here.

Including genotype data from case individuals from case-control studies made no appreciable difference to correlation estimates (supplementary fig. S4*A*, Supplementary Material online). Thus the bias in *r* that is predicted from theory when data derive from cases (supplementary text S3, Supplementary Material online) was not evident in our results.

### Role of Early-Life Mortality in Allele Frequency Clines

Since malaria-induced mortality occurs mainly during the first 2 years of life, it is possible that the observed correlation between malaria transmission intensity and the frequency of the *β^S^* allele, and other clinal alleles, might have been generated by bias in the sample caused by early-life malaria deaths rather than by historical differential selection by malaria within the subpopulations. However, the statistical test for an effect on the *β^S^* allele frequency of the age at which children were sampled (mean 7 months, SD 2.5) was not significant (*P* > 0.2 from a logistic regression model on individual allele data). Age effects were also absent (*P* > 0.2) for the other 56 candidate loci. The incidence of malaria during the period in which children were sampled for genotyping (2006–2010) was at its lowest for many years (supplementary fig. S1*A*, Supplementary Material online) with only two malaria deaths out of 6,814 (0.03%) hospital admissions of children under 1 year of age during the birth cohort sampling period versus 181 deaths out of 28,397 (0.64%) between 1989 and 2002. Combined, these results indicate that presampling bias at this or other loci is unlikely to be the cause of malaria-related clines in allele frequencies found here.

## Discussion

In this study, we applied ECA in a human population living under different malaria transmission intensities in a small geographic area in coastal Kenya in order to search for genes involved in resistance to malaria. We examined 57 candidate loci representing 39 genes that have been implicated in malaria pathogenesis: while 15 of these showed significant (*P* < 0.05) malaria-related clines in allele frequency, none of these reached significance when compared to a large set of randomly chosen loci and when population genetic structure in the background genome was controlled for in the analysis. This lack of strong signal by ECA among candidate loci accords well with the findings from large multisite case-control studies for severe malaria in which only a few of the 57 candidate loci selected by MalariaGEN for the first phase of rigorous testing have survived genome-wide tests for significance and replicability across populations (Jallow et al. 2009; Band et al. 2013; MalariaGEN 2014). Thus our results here, derived using independent methodology, help to negatively validate most members of this initial panel of malaria resistance candidate genes and thereby reprioritization of the set of genes taken forward into functional studies. Given that these candidates were selected on the basis of their membership of immune regulatory pathways associated with malaria disease severity, which are many and complex, or the set of receptors used by the parasite to invade red blood cells or bind to host cells, which are also many, their failure to yield strong associations with malaria in this and other large studies is perhaps not unexpected.

In contrast, our tests of nearly 10,000 SNP loci selected at random from across the genome revealed multiple loci with highly significant (*P* < 0.01) environmental correlations with malaria. Two of these (*CDH13* and *HS3ST3B1*) have previously been shown to be malaria-associated and both are involved in glycoprotein-mediated cell-adhesion pathways that are widely implicated in the pathogenesis of malaria. Given that there are approximately 20 malaria-associated genes that have been confirmed after stringent genome-wide testing and large-scale replication (Timmann et al. 2012; Band et al. 2013; MalariaGEN 2014; MalariaGEN et al. 2015), of which three were included in our survey of random loci (see Results), it is highly improbable that we could have obtained this result by chance alone. Moreover, seven of the 40 other genes among the top 1% of loci, are involved in cadherin-mediated adhesion and signaling at cell-cell junctions in the brain and/or vascular endothelium (*MAGI2*, *FN1*, *CTNND2*, *NRP2*, *NRXN3*, *RGS6*, *ADGRL2*). These results are further supported by the significant enrichment in closely related pathways among the top 10% of genes. Our results thus provide proof-of-principle that ECA has utility in the detection of novel malaria resistance genes through screens of large random sets of loci, and suggest that the cadherin-catenin complex operating in the brain or other vascular endothelium may play a central role in the pathogenesis of severe malaria.

Cadherins form the bridge between cells and are thus key molecules in development and tissue repair. While they are mostly known for their activity in neural cells, they also operate in the vasculature, a system that shares many genetic pathways, differentiation mechanisms, signaling mechanisms, and cross-talk with the nervous system (Carmeliet 2003). The cadherins that are abundantly expressed in the vasculature, principally CDH5 and CDH13, have been shown to mechanically induce cell signaling in the vascular endothelium in response to changes in blood flow (Birukov 2009). When blood vessels become obstructed and flow is reduced, increases in shear stress and vessel wall stretch, in conjunction with oxidative stress, trigger a signaling cascade that entails activation of sodium, chlorine, and potassium ion channels, calcium influx, activation of protein C kinases and G-protein coupled receptors, induction of NF-κB transcription factors, altered expression of genes coding for cytoskeletal components, inflammatory responses, and cell adhesion, all pathways that were found to be enriched in the top 10% of loci here. The consequences of this cascade are rigidification of blood vessels, inflammation, and impairment of the endothelial barrier function. CDH13, in particular, has been implicated in this process (Liu, Li, et al. 2015). However CDH13, which is found in high abundance in the heart, is unusual in that it lacks the cytoplasmic tail that in other cadherins anchors it to internal cytoskeletal components such as actin via linker molecules, principally catenins. This suggests that CDH13 mediates alteration of the vascular endothelium through its extracellular signaling, rather than binding, activity. A role for cadherin in malaria disease severity fits well with our current understanding of the pathogenesis of cerebral malaria in *P. falciparum* in which it is believed that adhesion of parasite-infected cells causes slowing of blood flow and perhaps blocking of microcapillaries, leading to higher shear forces, oxidative stress and inflammatory responses that damage the vascular endothelium, compromise the blood brain barrier, and ultimately result in life-threatening disease (Storm and Craig 2014).

The other previously identified malaria-associated gene found among the top 1% of genes here, *HSTST3B1*, encodes an enzyme that modifies sulfation patterns of heparan sulfate (glucosamine), a glycoprotein that is expressed on the surface of various cell types in *Plasmodium*’s human and mosquito hosts (Sinnis et al. 2007). Sulfate modifications of heparan sulfate have been implicated in the migration of sporozoites from skin to liver and invasion of liver cells (Frevert et al. 1993; Pradel et al. 2002; Coppi et al. 2007), the invasion of merozoites into red blood cells (Xiao et al. 1996; Kobayashi et al. 2010), and the adhesion of host cells to the PfEMP1 proteins that are expressed by the parasite on the surface of the infected red blood cell (Rowe et al. 1994; Barragan et al. 2000), a process that is associated with the most severe form of the disease, cerebral malaria (Carlson et al. 1990; Kaul et al. 1991; Rowe et al. 1995). Thus our finding fits well with the known association of heparan sulfate in moderating malaria pathogenesis. A recent study in West Africa found strong associations between variants in the exons of this gene and malaria parasitemia measured over a 2-year period (Atkinson et al. 2012). The exact link between sulfation patterns of heparan sulfate, the strength of binding of parasite-infected cells to host cells and downstream pathology is not yet well understood.

Among the candidate malaria resistance loci tested here, SNP rs2227478 in the *IL22* gene showed the strongest ECA signal. Although IL22 is generally known as an actor in the proinflammatory innate immune response to infection, relatively little is known about its role in relation to malaria. Recent evidence from the mouse malaria model *P. chabaudi* suggests that it may protect against liver damage (Mastelic et al. 2012). Genetic association studies that have tested multiple SNPs in this gene have failed to find significant effects on malaria disease severity (Dewasurendra et al. 2012; Apinjoh et al. 2013; Maiga et al. 2013; MalariaGEN 2014).

A malaria-related cline in the *β^S^* allele from the *HBB* locus has previously been reported in northern Tanzania in a set of nine villages located at different altitudes in three adjacent mountain ranges spanning 350 km in which there was evidence of regular genetic mixing from genotype data on 15 neutral loci (Enevold et al. 2007). In our study, we found a significant positive cline in *β^S^* across a malaria transmission intensity gradient but only when not allowing for genetic variation between populations in the background genome, as was also the case in the study from Tanzania. Replication of this malaria-related cline in *β^S^* across two independent studies conducted within small geographic regions with subtle geographic differences in allele frequency suggests that “the malaria hypothesis” may be operating on a fine geographic scale.

The B-determining allele at the *ABO* locus showed a positive environmental correlation with malaria that was significant (*P* < 0.05) without adjusting for population structure. This finding contrasts with the results from most case-control studies which have shown that, when combined with the A allele (i.e., in AB heterozygotes), this allele renders susceptibility to malaria (Fry, Griffiths, et al. 2008; Panda et al. 2011; MalariaGEN 2014). The possible reasons for this discrepancy are discussed below.

In addition to the genes in the cell-cell adhesion cadherin-catenin, and related pathways, two regulators of alternative splicing (*RBFOX1* and *SRPK2*) in neurons and the vasculature (Nowak et al. 2010) showed very strong environmental correlations with malaria. We suggest that these two genes, in addition to cadherins and catenins that are expressed in the vasculature, represent new candidate malaria resistance genes.

### Correspondence between ECA and Case-Control Studies

We found weak but significant correspondence between environmental correlations and the direct measures of protection against malaria from a case-control study (fig. 3B). This indicates that ECA can be used to help validate findings from case-control studies, and vice versa. Indeed, a hybrid ECA-case-control approach, as outlined in supplementary text S3, Supplementary Material online, could be applied to existing genotype data from case-control studies in order to capitalize on the already large investment in genome-wide searches for malaria-protective genes (MalariaGEN 2008, 2014).

It is striking that two SNPs with the strongest protective effects by case-control methods stood out as having negative associations with malaria by ECA (fig. 3B). The most striking example of this was the *ABO* SNP rs8176746 that determines the B versus A allele which showed a positive malaria-related cline with the B-producing allele in this study but in most case-control studies (supplementary table S2, Supplementary Material online), including that here, is reported to confer slightly higher or equal susceptibility of B carriers relative to A carriers, and considerably higher susceptibility of AB heterozygotes compared with A or B (Fry, Griffiths, et al. 2008; Panda et al. 2011; MalariaGEN 2014). The second example was the O-determining allele at the *ABO* locus coded for by SNP rs8176719 which has been very consistently demonstrated to protect against severe malaria relative to non-O alleles (A or B) in multiple case-control studies (Cserti and Dzik 2007; Fry, Griffiths, et al. 2008; Rowe et al. 2009; MalariaGEN 2014) but which showed a negative, albeit nonsignificant environmental correlation with malaria here (*r* = −0.23, *P* = 0.35).

Indeed, despite the fact that the concordance between results from ECA and case-control studies based on a large number of loci was significant (*P* < 0.001) it was, nonetheless, weak. The first potential explanation for this relates to the design of case-control studies. Typically in malaria genetic epidemiology studies, because of the method of recruitment of cases, the genetic make-up of cases and controls differs (Band et al. 2013). Not only can this readily lead to false associations for loci that confer no protection (Price et al. 2010), but it is expected to cause systematic negative bias for protective alleles if, as our study illustrates, there are positive geographic associations between the frequency of the protective allele and the amount of disease transmission in the local population. Using the B allele at the *ABO* locus to illustrate, in case-control studies, sampling across the study population without rigorous matching for location would generate higher frequencies of the B allele malaria among cases than controls because cases are more likely to derive from high malaria transmission areas. This would lead to the interpretation that the B allele confers disease susceptibility, in contrast to the implied protective effect by ECA. We suggest that some of the previous inconsistencies in results between different case-control studies might be resolved once more attention is paid to the fine-scale population substructure and heterogeneity in disease transmission intensity that appears to be typical of malaria case-control study sites in Africa. We further suggest that the development of a hybrid statistical “ECA-case-control” model that accounts for transmission-related clines in allele frequencies while simultaneously estimating relative disease risk in cases versus controls will improve both the power and reliability of detection of disease-modifying alleles through the joint use of multiple, independent and complementary types of information.

Another possibility for low concordance between results from case-control and ECA methodology is that resistance-conferring alleles are under balancing selection by malaria on the one hand and negative selection by another force. Indeed, balancing selection is the basis for the “fine-tuning” model on which ECA is predicated. Balancing selection can arise within a locus through negative pleiotropy whereby the allele protects against one disease but confers susceptibility to another. It can also be mediated by a second locus through negative epistasis where alleles at different loci interact to counteract their individual protective effects. The case of the O allele at the *ABO* locus may be an example of within-locus balancing selection: whereas the O allele clearly protects against malaria, non-O alleles may protect against other diseases caused by viruses and bacteria (Anstee 2010) which show strong comorbidity with malaria (Scott et al. 2011; Church and Maitland 2014). If this is true, case-control studies for a single disease in the presence of the other disease would lead to underdetection of a protective effect. In the case of two-locus balancing selection (negative epistasis), for which there is a clear example in the malaria-associated *HBB* and *HBA* loci (Williams et al. 2005; May et al. 2007), signals of protective effects from case-control studies for individual loci are likewise expected to be obscured. Indeed, the finding of a strongly discordant result between case-control and ECA studies for a given locus may flag the existence of competing selective forces, either from different diseases or from interactions with alleles at the same locus or from different loci acting on the same disease, thereby potentially stimulating new hypotheses regarding mechanisms of protection of these polymorphisms.

A third source of bias in case-control studies is that selection by malaria in early life will enrich the more highly exposed case population with resistance alleles, thus reducing or even reversing estimates of protective efficacy. In contrast, early life protection will enhance signals of protection obtained by ECA. This may well have been the case in the study from northern Tanzania by Enevold et al. (1987) in which samples for genotypes were collected from children less than 5 years, the age-group in which most malaria mortality occurs. In our study, we could rule out this explanation because of the unusually low incidence of malaria and malaria-related deaths during the recruitment period and because most samples were collected at a very early age. In most malaria case-control studies, however, early-life malaria deaths are likely to be substantial prior to the age of collection of samples for genotyping. If ECA were to be retrospectively applied in these populations, results from ECA and case-control studies are expected to be less concordant than found here.

Discrepancies between studies may also arise because the SNPs under analysis are not causative, but are instead markers in linkage disequilibrium with the causative mutation and thus potentially differ in phase between populations. Advances in genotype imputation and other statistical methods that allow combining of information from multiple SNPs per gene and across studies are helping to address this issue (Marchini et al. 2007; Band et al. 2013) and could be adapted for use in ECA.

In addition to the biases in ECA and case-control studies discussed above, the fact that both designs yield correlative rather than causative results cautions against their overinterpretation. For example, it could be argued that higher frequencies of the protective alleles in high transmission areas are the cause, rather than the consequence, of malaria deaths. However, we feel this is unlikely in this case given that even the most malaria-protective allele (*β_S_*) accounts for less than 2% of the variation in malaria incidence in the general population (Mackinnon et al. 2005), with ecological factors that drive mosquito abundance, rather than genetics, being the primary determinant of disease risk.

### Implications

Our results demonstrate, using malaria as an example and a subset of all genes in the genome, that ECA can be used to detect adaptive genes in relatively stable indigenous human populations, even with a small geographic area. They further lend general support to the proposal that much of human adaptation to diverse selective environments involves subtle shifts in allele frequencies of genes under balancing selection rather than strong selective sweeps involving directional, dramatic allele frequency changes (Pritchard et al. 2010). If balancing selection is more prevalent than commonly assumed, this would have wider implications for understanding the maintenance of quantitative genetic variation (Hill et al. 2008). It would also have practical implications for genome-wide search strategies for adaptive genes: if human evolution proceeds mainly through balancing selection, then one of the commonly used strategies—scanning the genome for conserved haplotypes as signatures of recent hard selection—may detect only a fraction of the targeted genes. Likewise, for the reasons discussed above, the widely used case-control methodology for genome-wide searches may, in the case of infectious diseases, suffer from bias arising from geographic confounding between allele frequencies and disease exposure. ECA offers a number of distinct advantages over traditionally used methods, especially in settings where linkage disequilibrium is low and genetic diversity and environmental heterogeneity are high, as in Africa. We propose, therefore, that ECA be incorporated as an additional tool into genome-wide case-control studies for disease resistance loci. It can be easily implemented, at no extra cost, whenever the disease-capture methodology, for example, hospital-based surveillance, collects information on the patient’s location of residence. Results from ECA and case control approaches are likely to be complementary, cross-validating and more informative with regard to the potential mechanisms by which individual protective alleles and their interacting partners affect disease outcomes.

The possibility that the environmental correlations we observed were driven by historical admixture across a geographical malaria gradient cannot be totally dismissed. Archeological and anthropological evidence indicate that the collection of ethnic groups in our study area, known as the Mijikenda, a grouping of nine culturally related though distinct groups, have been living in close association with one another since their joint migration 400 years ago from southern Somalia to their present location where they have been settled since (Spear 1974, 1977; Rosa 1987) with, perhaps a migration of their forebears from northern Tanzania a millennium before that (Rosa 1987; Spear 2000). There is also evidence that this migration of the Mijikenda group along the Somalia–Kenya–Tanzanian coastal hinterland has involved several distinct migratory events and settlement of subpopulations of the Mijikenda in different locations (Spear 1974; Spear 2000). For example, while the Chonyi and Giriama subgroups of the Mijikenda are recorded as migrating to the area 400 years ago, this migration did not include the Kauma (Spear 1974), thus explaining their distinct genetic makeup as observed here (supplementary fig. S2, Supplementary Material online). Today, the descendants of these sets of migrants still maintain their ethnicity, both culturally and genetically, as confirmed here by genotyping, and, furthermore, tend to occupy areas of different malaria transmission intensity across the north–south transect of our study area. This has led to geographic patchiness in ethnic group overlaid by patchiness in malaria transmission. Such patterns of ethnic grouping by ecological type echo a general feature of populations of Kenya and Tanzania as revealed by dermatoglyphic studies (Rosa 1985, 1987), and are likely to be a feature of many other populations in Africa. Thus genetic association studies, whether by case-control or ECA, need to ensure that fine-scale population structure is adequately captured in their design and analysis.

We have proposed that the malaria-related frequency clines observed here are caused by balancing selection on genes that are directly selected by survival of malaria. It is also possible, however, that the clines were generated by indirect selection on a genetically determined behavioral trait such as propensity to migrate to less disease-ridden areas or to alter the environment in a way that reduces exposure to the disease. For example, the least malaria-resistant ethnic group in this study—the Giriama—may have chosen, historically, to settle in areas of lower malaria transmission intensity, or to change their farming practices, forestation levels, or lifestyle in order to limit their malaria exposure. Humans are consummate niche constructors, and much of their adaptation to diverse habitats has been attributed to their ability to alter their environment, and hence evolutionary trajectory, through cultural as well as genetic inheritance (Laland et al. 2010). Selection through such indirect means will, as for direct selection on survival, leave spatiogenetic signatures in populations experiencing different selective environments thus further expanding the prospects of finding genes that confer protection against disease using ECA.

## Materials and Methods

### Ethics Statement

The study was approved by the KEMRI/National Ethics Review Committee. The parents of all study participants provided written informed consent for both blood sampling and genotyping.

### Study Design

The primary aim of this study was to test for “environmental correlations” between the population frequencies of polymorphic alleles in 57 candidate malaria resistance loci and historical levels of malaria transmission intensity in sets of geographically divided subpopulations. To achieve this, environmental correlations were computed between estimates of subpopulation allele frequencies obtained from 12,425 children under 1 year of age in a genetics birth cohort, and their corresponding subpopulation malaria prevalence estimates calculated from 82,042 records on malaria slide positivity from children less than 15 years of age recruited at the hospital or in the community over a 50-year period. Environmental correlations for the 57 candidate loci were compared with those from a control set of 9,756 putatively neutral SNPs genotyped in a subset of individuals from the birth cohort (table 1).

Genotypes were also obtained from case individuals from two case-control studies, one for severe malaria (MalariaGEN 2014) and one for bacteremia (Rautanen A, unpublished data). Environmental correlations were computed using three different genotype data sets (control individuals, including bacteremia cases, malaria cases only, and all cases and controls combined); three independent estimates of malaria prevalence data (from hospital admissions in 1989–2002, from hospital admissions in 2003–2010, and from community surveys in 1960–2007); and three levels of population subdivision (11, 15, and 38 subpopulations) (table 1). Unless stated otherwise, results on environmental correlations presented in the figures are based on malaria prevalence computed from combined hospital and community data across all years, genotype data from all control and case individuals, and 11 subpopulations.

### Study Area

The study was conducted in an area of coastal Kenya spanning approximately 890 km^2^ which is served by the Kilifi District Hospital and which is under surveillance by the Kilifi Health and Demographic Surveillance System. This area is divided for administrative purposes into “locations” and “sublocations,” termed “subpopulations” here (fig. 1A). A main road runs north-south through the area, a major waterway (Kilifi Creek) runs east-west and the hospital is located at the intersection of these geographic features near the center of the area. The population comprises three main ethnic groups—the Chonyi, Giriama**,** and Kauma—which constitute a subset of nine loosely defined ethnic groups known as the Mijikenda who have occupied the Kenyan coast for the past 400 years after migrating together from south Somalia (Spear 1974, 1977). The Kauma typically reside in the center and west of the study area, the Giriama in the north and the Chonyi in the south (fig. 1 and supplementary fig. S2, Supplementary Material online).

### Genotyping

The primary analysis was conducted on data from 10,597 children born within the study area between August 2006 and September 2010 who form a birth cohort under investigation for genetic susceptibility to infectious diseases (the “Kilifi birth cohort”) (Williams et al. 2009). DNA was extracted from capillary blood samples collected on cohort members at recruitment between 3 and 12 months of age. Children in the birth cohort were genotyped for 57 SNPs representing 39 candidate malaria resistance loci (supplementary table S1, Supplementary Material online) selected on the basis of their showing malaria protection in previous studies or for their known role in red blood cell physiology, receptors for parasite binding, or the immune response (MalariaGEN 2014). Genotypes were generated using the Sequenom iPLEX platform for all loci except the *HBA* that codes for α−globin for which the -α^3.7^ deletion, the most common cause of the African form of α^+^-thalassemia, was genotyped by PCR (Chong et al. 2000).

To form a putatively neutral comparison set, genotypes for 10,000 SNPs chosen at random from across the genome, genotype data were generated on 5,214 children from two case-control studies, one for severe malaria, and one for bacteremia. Control individuals for these studies were members of the birth cohort described above and so were sampled for DNA before 1 year of age whereas case individuals were sampled at the time of disease. SNP genotypes for case-control individuals were obtained from the Illumina Omni 2.5M and the Affymetrix 6.0 platforms for the malaria and bacteremia studies, respectively. The 10,000 SNPs chosen for analysis were a random subset of those that were included on both chips and which were no less than 0.1cM apart in the HapMap combined recombination map. SNPs that fell within the 57 candidate malaria loci, and those that showed allele frequency differences of greater than 0.1 between the two platforms on the overlapping set of samples, were excluded, leaving 9,756 SNPs for the final analysis. These represented 3,010 protein-coding genes and 1,022 noncoding RNA genes: the remainder fell within intergenic regions. Ancestral alleles, global allele frequencies and other information on each SNP were retrieved from the dbSNP database (dbSNP 2015) using the rsnps package in R (Chamberlain and Ushey 2015).

### Malaria Prevalence

Indices of long-term malaria transmission intensity were obtained from malaria slide positivity data from hospital admissions and from community surveys conducted by the Kenyan Ministry of Health. Hospital data were based on all patients less than 15 years of age who were admitted to Kilifi District Hospital between 1989 and 2010. Because of minor changes to the administrative boundaries of locations that took effect in December 2002, these estimates were calculated separately for the periods May 1989 to December 2002 (*N* = 43,037) and January 2003 to September 2010 (*N* = 19,282), a period during which transmission intensity was considerably lower (fig. 1D, supplementary fig. S1, Supplementary Material online). For the first period, the data were divided among 11 subpopulations: in 2003, three of the administrative areas thus dividing into 15 subpopulations for the second period. These 15 were further divided based on administrative boundaries into 38 subpopulations.

To address the issue of potential bias in subpopulation-specific malaria prevalence estimates from hospital admissions data arising from differences in distance to the hospital or other factors that affect health-seeking behavior, a third index of malaria prevalence was obtained from 216 independent community-based surveys on 20,331 children conducted between 1960 and 2007 in the study area by the Kenyan Ministry of Health and the KEMRI-Wellcome Trust Research Program (Snow et al. 2015). Records were categorized into the same 11 subpopulations as for the hospital admissions data from 1989 to 2002, excluding subpopulation Gede for which there were no records.

To obtain best estimates of malaria prevalence by subpopulation, means were adjusted for uneven sampling across years and subpopulations by fitting a binomial regression model with fixed-level factors of subpopulation, year, and source (hospital vs. community) as terms in the model. Predicted means and standard errors for each subpopulation, standardized to the median year in the data (2003 for when all hospital and community data were combined), were obtained from the fitted model and used in analyses for malaria-related clines in allele frequencies described below. Estimates were obtained for each of the three malaria prevalence data sets separately, standardized to 1997 and 2006 for data on hospital admissions from 1989 to 2002 and 2003 to 2010, respectively, and to 1995 for community survey data from 1960 to 2007, thus providing three independent environmental variables to test for robustness of the ECA results.

### Malaria-Related Clines in Allele Frequencies

To test for a relationship between malaria transmission intensity and the frequencies of malaria resistance alleles across geographically separate subpopulations, we calculated subpopulation-specific allele frequencies for all 57 candidate loci using genotype data from all children in the birth cohort and then related these to subpopulation-specific malaria prevalence in the wider population. This was done, first, using binomial logistic regression analysis and, second, using a Bayesian method to fit a population genetics model of allele frequency variation between populations that takes into account both the genetic relatedness among populations and error in frequency estimates that arises from small samples (see below).

The logistic regression model was fitted to binary data on individual alleles obtained from genotype data (i.e., two records per child) with subpopulation-specific malaria prevalence estimates fitted as a linear covariate. The reference allele in the analysis was the ancestral allele (supplementary table S1, Supplementary Material online): thus the results presented here describe the relationship between malaria prevalence and the derived allele. Regression coefficients from the logit scale were back-transformed for the purposes of plotting the predicted shape of the malaria-related cline on the original scale. Estimates were tested for significance by two-tailed *t*-test. A second model was fitted with a single fixed-level effect for subpopulation in order to determine whether subpopulations varied significantly in allele frequency irrespective of malaria prevalence. A third model was fitted with subpopulation as a random effect in order to obtain estimates of the between and within subpopulation variances in allele frequency. These models were fitted using the glmer package in R (Bates et al. 2015). The amount of between-population to within-population variance in allele frequencies was estimated using the likelihood ratio based pseudo R-squared statistic implemented in the r.squaredLR command in the MuMIn package in R (Bartoń 2015).

However, the logistic regression model described above does not take into account the genetic relationships between subpopulations that might arise in neutral alleles as a result of shared population history and gene flow, and the error measurement in allele frequencies, which might bias the correlations between allele frequencies and malaria prevalence (Coop et al. 2010). Therefore we used the “Bayenv” method of Coop et al. (2010) in which a null model of multivariate normal population allele frequencies, with covariance between populations, is assumed. This null model is used as the background against which a test for a relationship between allele frequencies and an environmental variable of interest is made. By fitting this model in a Bayesian framework, the level of statistical support for an environmentally related cline in allele frequency against the distribution of clines under the null model, while simultaneously accounting for genetic population structure, is obtained. In a later version of the Bayenv method (Gunther and Coop 2013), statistical tests are performed on “standardized” population allele frequencies in order to reduce the effects of outliers. Here, we used the latter method as implemented in the “Bayenv2.0” package (Gunther and Coop 2013) to calculate the Pearson correlation, *r*, for each of the 57 candidate loci and the 9,756 random loci. Empirical *P* values for the 57 candidate loci were computed from the null distribution of *r* values for the random loci. Since there were considerably fewer genotype data for control loci than candidate loci (table 1), thus causing higher sampling variance of allele frequencies and smaller correlations among control loci than candidate loci, *r* for candidate loci was computed from random subsamples of the full data set with N equal to the number of genotypes available for the random SNP loci. This was repeated 100 times, and the median values of *r* and its empirical *P* value were taken to be the best estimates. “Asymptotic *P* values” for *r* were also computed using the Student’s *t*-test method for Pearson correlations with degrees of freedom equal to the number of subpopulations minus two. This method assumes that the subpopulation allele frequencies are estimated without error and thus provides conservative *P* values for *r.*

To allow for multiple testing in the candidate loci, “global” test statistics were computed for the 57 candidate loci combined and compared with the distribution of this statistic from 1,000 random draws of 57 loci from the pool of 9,756 random SNP loci plus 57 candidate loci. The global statistics used here were, first, the sum of the absolute values of *r* and, second, the sum of the log_10_ empirical *P* values for *r*. As above, due to the fewer genotypes among random loci than candidate loci, global test statistics for candidate loci were based on 100 replicate subsamples of the full genotype data set. Global test statistics were also computed for the six loci showing the strongest correlations out of the set of 57 in each draw.

Comparisons between candidate and random loci were performed using data from all 9,756 random loci except when some subpopulation frequencies were zero due to small sample size, as occurred for loci with low allele frequencies, and when the population was divided into 38 subpopulations, in which case the locus was not included in the random SNP set. To check whether differences in the distribution of allele frequencies between candidate and random loci affected significance tests, for each candidate locus, a subset of 70–80 random SNP loci that had allele frequencies within 0.02 of the candidate locus, were selected to form a total subset of 4,082 loci which was then used to form the empirical distribution for significance testing. Results using this distribution were compared to those when the full set of random SNPs were used for the empirical distribution.

### Comparison with Case-Control Results

To determine whether signals of protection against malaria by ECA corresponded with those directly measured by case-control methods, for each SNP, allele count data from all control individuals (i.e., the full birth cohort, *N* = 10,597) and all malaria cases were analyzed for differences in frequencies between cases and controls by logistic regression under a model with a fixed effect for cases versus controls, a linear covariate for malaria prevalence (using the output of standardized values from the Bayenv2 package), and an interaction between these. A reduced model containing only the case-control effect was also fitted. Estimates of the case versus control effect and estimates of the differences in slope between cases and controls (the interaction effect), were regressed on *r* values from ECA and tested for significance based on the Spearman rank correlation. This nonparametric test was used in order to avoid excessive influence of loci with very strong protective effects, such as *HBB*.

Since frequencies of protective alleles are biased downwards among cases in case-control studies relative to overall population frequencies, it may be expected that ECA based on data from case-control studies would lead to biased estimates of *r*. Conversely, environmental correlations might lead to bias in estimates of protective effects in case-control studies if the environmental correlation is not taken into account in the sampling design and analysis. A theoretical analysis was therefore undertaken to determine the effect of case-control status on estimates of *r* (supplementary text S3, Supplementary Material online).

### Genetic versus Geographic Population Structure

To compare genetic distances with geographic distances, multidimensional scaling, performed using the cmdscale command in the stats package in R (R Core Team 2014), was applied to the subpopulation genetic variance-covariance matrix (dimension 11 × 11) estimated from genotype data on the 9,756 random loci data in the Bayenv package. This yielded a 2D representation of genetic distances among subpopulations. The genetic map was rotated and scaled to obtain the best fit to the geographic map using the procrustes function in the vegan package in R (Oksanen et al. 2015). Genetic clustering of subpopulations was visualized by hierarchical clustering using the hclust command within the R stats package (R Core Team 2014) and the pheatmap package (Kolde 2015).

### Gene Set Enrichment Analyses

To determine whether the genes with strongest environmental correlations were concentrated in specific molecular pathways, the genes represented among the top 10% of SNPs by ECA were subjected to overrepresentation tests using PANTHER (Mi et al. 2013), and network-based pathway enrichment tests using EnrichNet (Glaab et al. 2012). Reference sets used for comparison were all characterized and annotated genes among the 9,756 random SNPs (i.e., noncoding RNA genes were excluded) and which were found in the respective PANTHER and EnrichNet databases. Tests were made for gene sets based on gene ontologies using GO-Slim and KEGG terms, signaling pathway types using “PANTHER Pathways,” and protein domain types using InterPro.

## Supplementary Material

Supplementary figures S1–S4, tables S1–S3, and texts S1–S3 are available at *Molecular Biology and Evolution* online (http://www.mbe.oxfordjournals.org/).

Supplementary Data
