# Supplementary material for: Environmental Correlation Analysis for Genes Associated with Protection against Malaria
Source: Mol Biol Evol. 2016 Jan 6;33(5):1188–204. doi: 10.1093/molbev/msw004 (PMC4839215; doi:10.1093/molbev/msw004)
Supplement: Supplementary Data [file supp_33_5_1188__index.html]

Environmental Correlation Analysis for Genes Associated with Protection against Malaria — Environmental Correlation Analysis for Genes Associated with Protection against Malaria — Environmental Correlation Analysis for Genes Associated with Protection against Malaria — Supplementary Data 

# Environmental Correlation Analysis for Genes Associated with Protection against Malaria

## Supplementary Data

files

- Supplementary Data - zip file
