# Supplementary material for: Environmental Correlation Analysis for Genes Associated with Protection against Malaria
Source: Mol Biol Evol. 2016 Jan 6;33(5):1188–204. doi: 10.1093/molbev/msw004 (PMC4839215; doi:10.1093/molbev/msw004)
Supplement: Supplementary Data [file supp_msw004_suppl_data.zip › Supplementary Text 3 Mackinnon MBE-15-1092 Dec 2015.pdf]

Supplementary text 3.

Environmental correlations in case-control designs

Environment-related clines of protective alleles estimated from case-control data may be expected to differ from those estimated from an unselected population such as a birth cohort. This is because alleles that render people susceptible to disease are enriched among the case population compared to the control population. Indeed, these frequency differences are the basis for detecting protective alleles using case-control study designs. As shown below, depending upon the mode of inheritance of the protective allele, these frequency differences between cases and controls can affect environment-related gradients in allele frequency.

To compute the expected allele frequencies among cases and controls across an environment-related gradient in allele frequency we extend the model of Witte et al. (2014) as follows. Denoting p as the frequency of the protective allele, B, in the general population at birth, where protection is defined relative to the wild-type allele, b; the relative risks of disease for the three genotypes *bb*, *Bb* and *BB* as 1, R and Rd, respectively, where R is relative risk of disease given one copy of the protective allele (R<1); d as a dominance parameter (d=1 for complete dominance, d>1 for heterozygote advantage (over-dominance), and d=R for a multiplicative mode of protection for two copies of the allele, i.e., in homozygotes); f_0 as the absolute risk of disease among wild-type homozygotes, and K as the overall prevalence of disease in the population, the allele frequencies among cases and controls can be calculated from the genotype probabilities, conditional on disease status, as in the table below (Witte et al. 2014).

Table. Genotype by disease probabilities.

Genotype (G)	P(D|G)[a]	P(D)[b]	P(G|D)[c]
**bb**	f_0	$(1-p)^2 f_0$	$(1-p)^2 f_0/K$
**Bb**	f_0R	$2p(1-p)f_0R$	$2p(1-p)f_0R/K$
**BB**	f_0Rd	$p^2\ f_0Rd$	$p^2\ f_0Rd/K$
		Sum = K	

[a] P(D|G) denotes the probability of disease given the genotype.
[b] P(D) denotes the probability of disease. These sum to K, the population prevalence of the disease.
[c]P(G|D) denotes the probability of the genotype given the disease, i.e., the frequency of the genotype within the group with disease.

From these probabilities, the frequency among the group with disease (the cases) can be calculated as:

$$p_{cases} = \frac{pR(1+p(d-1))}{pR\big(1+p(d-1)\big)+(1-p)(1+p(R-1))} \qquad (1)$$

The frequency among controls can be calculated from the prevalence and general population frequency as:

$$p_{controls} = \frac{1}{1-K}(p - Kp_{cases}) \qquad (2)$$

After transforming to the logit scale, the relationship between allele frequencies in the case population as a function of frequencies in the general population, and hence the behaviour of the environment-related cline in frequency in cases relative to controls, can be modelled using the following regression equation:

$$\log\left(\frac{p_{cases}}{1-p_{cases}}\right) = \log\left(\frac{p}{1-p}\right) + \log(R) + \log\left(\frac{1+p(d-1)}{1+p(R-1)}\right) \quad (3)$$

Eq. (3) shows that when R=d (i.e., protection per allele is multiplicative), and assuming that R is a fixed value across the range of p and any environmental correlate of p, then logit allele frequencies in the case population are always a fixed value of log(R) lower than in the general population (Fig. 1). Thus an environment-related cline in p will be the same in cases as in the general population when R=d. Fitting a logistic regression model of p_{cases} on p to data from case-control studies would yield a slope of one and an intercept of log(R). Thus estimates of the direct protective effect of the allele in case-control studies would not be biased by the presence of environmental correlations, and *vice versa,* if the alleles act multiplicatively on disease risk on the observed scale (i.e., additively on the log risk scale).

However, when d>R (i.e., when there is dominance), the regression slope of p_{cases} on p is greater than one and the intercept (i.e., when p = 0.5) diminishes in magnitude compared with when R=d. It nonetheless remains negative (i.e., reflects a protective effect) as long as d>1/R, i.e., the *BB* homozygote is more protected than the bb homozygote. Under a model of perfect overdominance (d=1/R, i.e., same levels of protection in both homozoygotes), the frequency among cases is lower than in the general population for p < 0.5 and higher in the general population for p > 0.5. Thus dominance causes positive bias in environmental correlations among cases, and negative bias in protective effects from case-control studies, if not allowed for in the ECA and case-control analyses.

The bias in allele frequencies among cases relative to the control population described above has several implications for ECA of data from case-control studies. First, if data from cases and controls are pooled for the purposes of ECA, and if case-control status is ignored, the extra variance in p caused by systematic differences between cases and controls would weaken any correlations between p and the environmental variable thereby eroding power. Second, if the data were analysed under a framework that incorporated both case-control status and environmental clines (under the model in Eq. (3)), a finding of differences in slope between cases and controls could provide information on the mode of inheritance. Thus simultaneous estimation of direct protective effects and allele frequency clines using data from case-control studies using the framework described here would maximise the amount of information obtained from the data while minimising bias in estimates of the protective effects and environmental correlations.

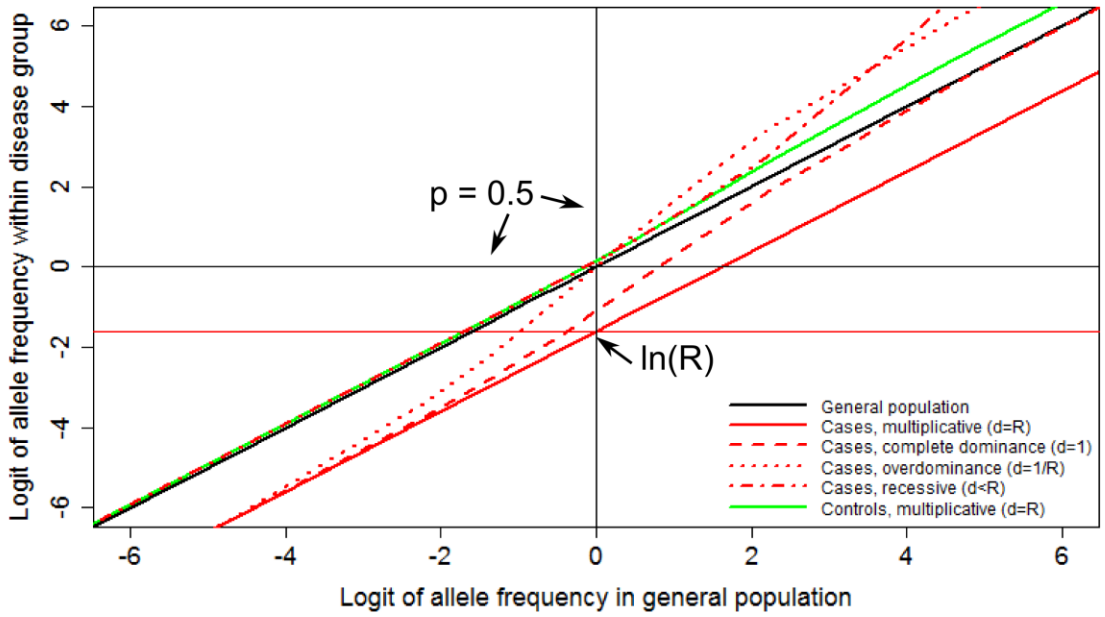

Fig. 1. Relationship between frequency of the protective allele in cases compared with the general population. Under a multiplicative model on the observed scale, the logit transformed allele frequency among cases (the diseased group) (y-axis) increases proportionally with logit transformed allele frequency in the general population, *p* (x-axis) and hence also with an environmental correlate of *p*. Under this multiplicative model, a fixed deficit of log(R) in frequency between cases and controls across the range of frequencies is maintained (red solid line cf. black solid line) but the environmental correlation remains the same in cases as in controls. As the protective effect in heterozygotes increases relative to that in homozygotes ($d>R$), i.e., there is dominance, the magnitude of this frequency deficit is reduced and the ratio of allele frequencies in the case population relative to the general population increases, thus increasing the correlation with an environmental correlate of *p* (red dashed line vs. black solid line). Thus under some conditions, clines in allele frequency across an environmental gradient are expected to be different in cases compared to those in the general population thus causing bias in ECA using data from case-control studies. Values shown are for R=0.2 and K=0.1.

Reference

Witte, JS, PM Visscher, NR Wray. 2014. The contribution of genetic variants to disease depends on the ruler. *Nat Rev Genet* 15:765-776.
