# Supplementary material for: Environmental Correlation Analysis for Genes Associated with Protection against Malaria
Source: Mol Biol Evol. 2016 Jan 6;33(5):1188–204. doi: 10.1093/molbev/msw004 (PMC4839215; doi:10.1093/molbev/msw004)
Supplement: Supplementary Data [file supp_msw004_suppl_data.zip › Supplementary Material - Figures Mackinnon MBE-15-1092 Dec 2015.pdf]

Supplementary Figures

For “Environmental correlation analysis for genes associated with protection against malaria”

Margaret J Mackinnon, Carolyne Ndila, Sophie Uyoga, Alex Macharia, Robert W. Snow, Gavin Band, Anna Rautanen, Kirk A. Rockett , Dominic P. Kwiatkowski and Thomas N. Williams, in collaboration with the MalariaGEN Consortium

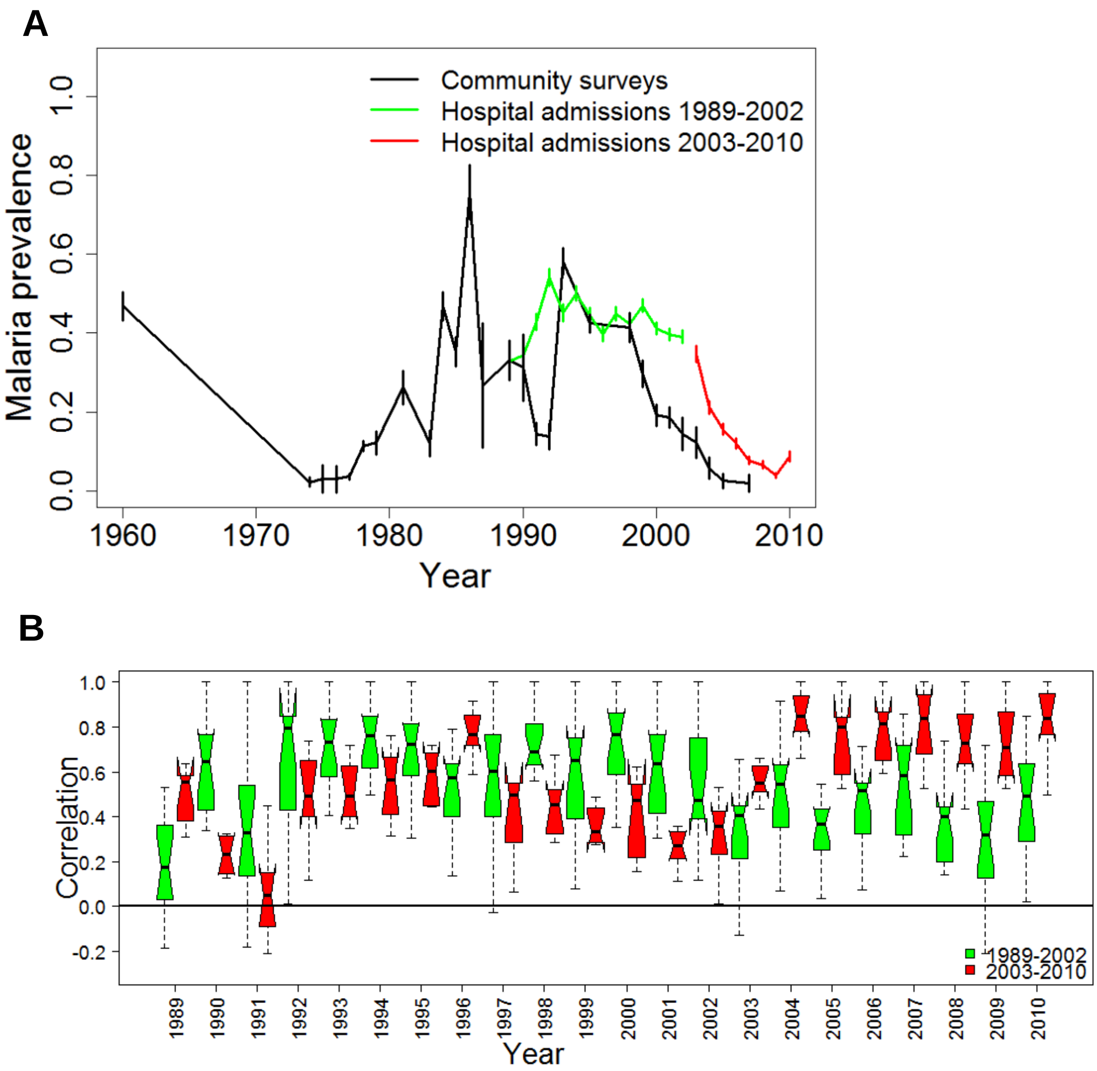

Fig. S1. Malaria prevalence and stability of spatial variation through time in the study population.

(*A*) Mean malaria prevalence (y-axis) through time (x-axis) in the study population. Values plotted are estimates from a logistic regression model fitted to malaria slide positivity data among children <15 years of age admitted to Kilifi District Hospital (green and red lines, see legend) or from community surveys (black lines) with subpopulation as a fixed-level factor in the model in order to adjust for unequal sampling across the study area over the years. Values are adjusted to the mean prevalence in Tezo which is the subpopulation with most records across all years. Vertical bars indicate 95% confidence intervals around the means. (*B*) Between-year correlations in malaria prevalence across subpopulations (y-axis). Each pair of boxes within year (x-axis) represents the distribution of correlations between the year shown on the x-axis and every other year, separated according to the time period (1989-2002 vs. 2003-2010) in which the latter occurred (green vs. red, see legend). Box boundaries represent interquartile ranges, whiskers extend to 1.5 times these, and notches indicate approximate 95% confidence intervals. Most between-year correlations were significantly above zero, the expected value if spatial variation in malaria prevalence changed randomly through time. Correlations within time periods tended to be stronger than between periods.

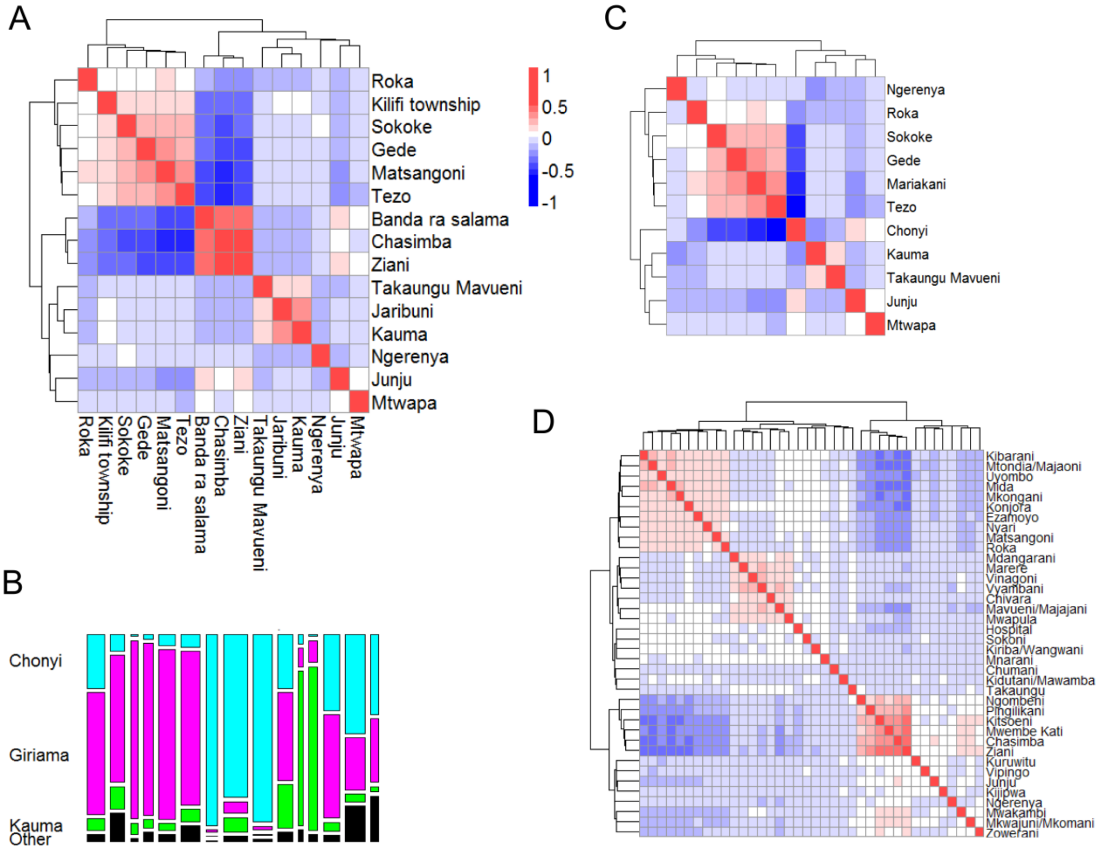

Fig. S2. Genetic structure of the study population.

(*A*) Heatmap representation of genetic correlations among 15 subpopulations based on 9,756 random SNP loci. The genetic correlation matrix was estimated by computing across subpopulation correlations in frequencies of SNP loci. Red and blue represent positive and negative correlations, respectively (see legend in (*A*)). Dendrograms were derived from hierarchical cluster analysis. (*B*) Proportions of each of the three dominant ethnic groups (Chonyi, Giriama and Kauma, rows) and other ethnic groups in each of the 15 subpopulations columns). Columns are ordered the same as in (*A*). (*C*) As for (*A*) but for 11 subpopulations. (*D*) As for (*A*) but for 38 subpopulations. The 15 subpopulations were created from the 11 subpopulations in 2003 when administrative boundaries changed as follows: Chonyi was subdivided into Banda ra Salama, Chasimba and Ziani, Kauma was divided into Jaribuni and Kauma, and Tezo was divided into Kilifi township and Tezo. The 38 subpopulations are subdivisions of the 11/15 populations.

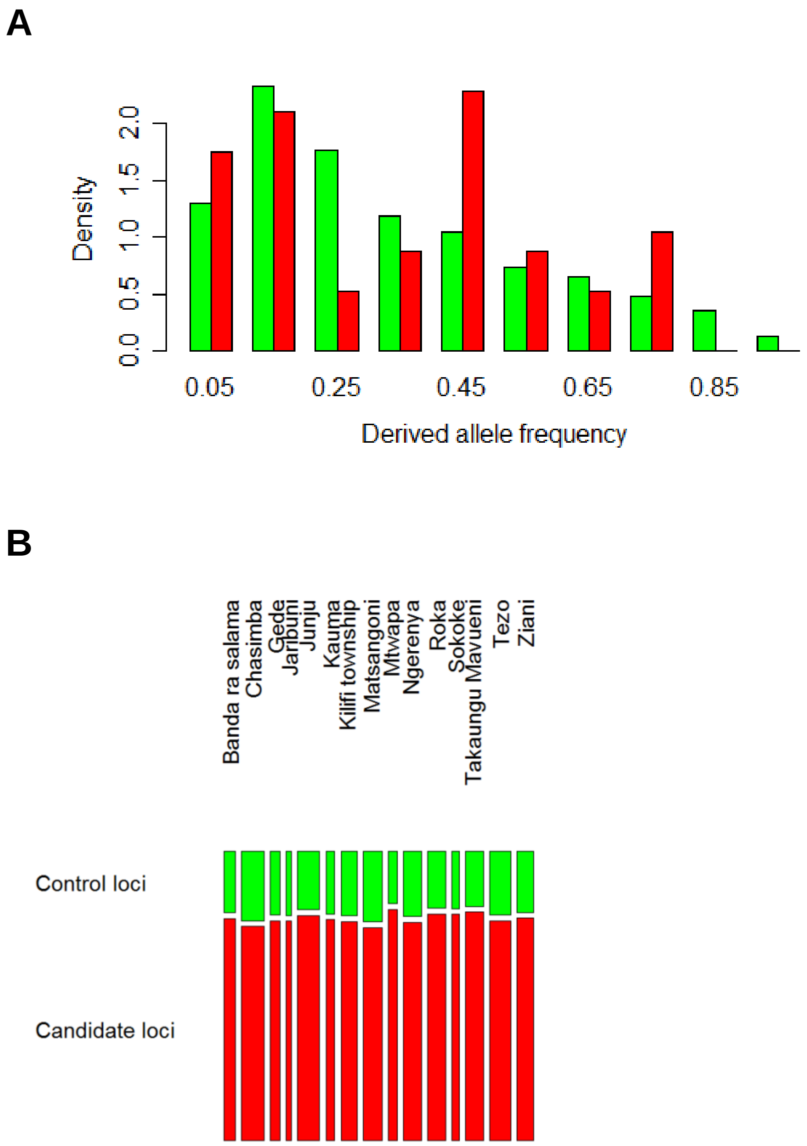

Fig. S3. Distributions of minor allele frequencies and numbers of observations by subpopulation for candidate and random loci.

(*A*) Distribution of derived allele frequencies for 57 candidate loci (red) and 9,756 random loci (green). (*B*) Relative numbers of observations by subpopulation for the 57 candidate loci (red) and the 9,756 random loci (green).

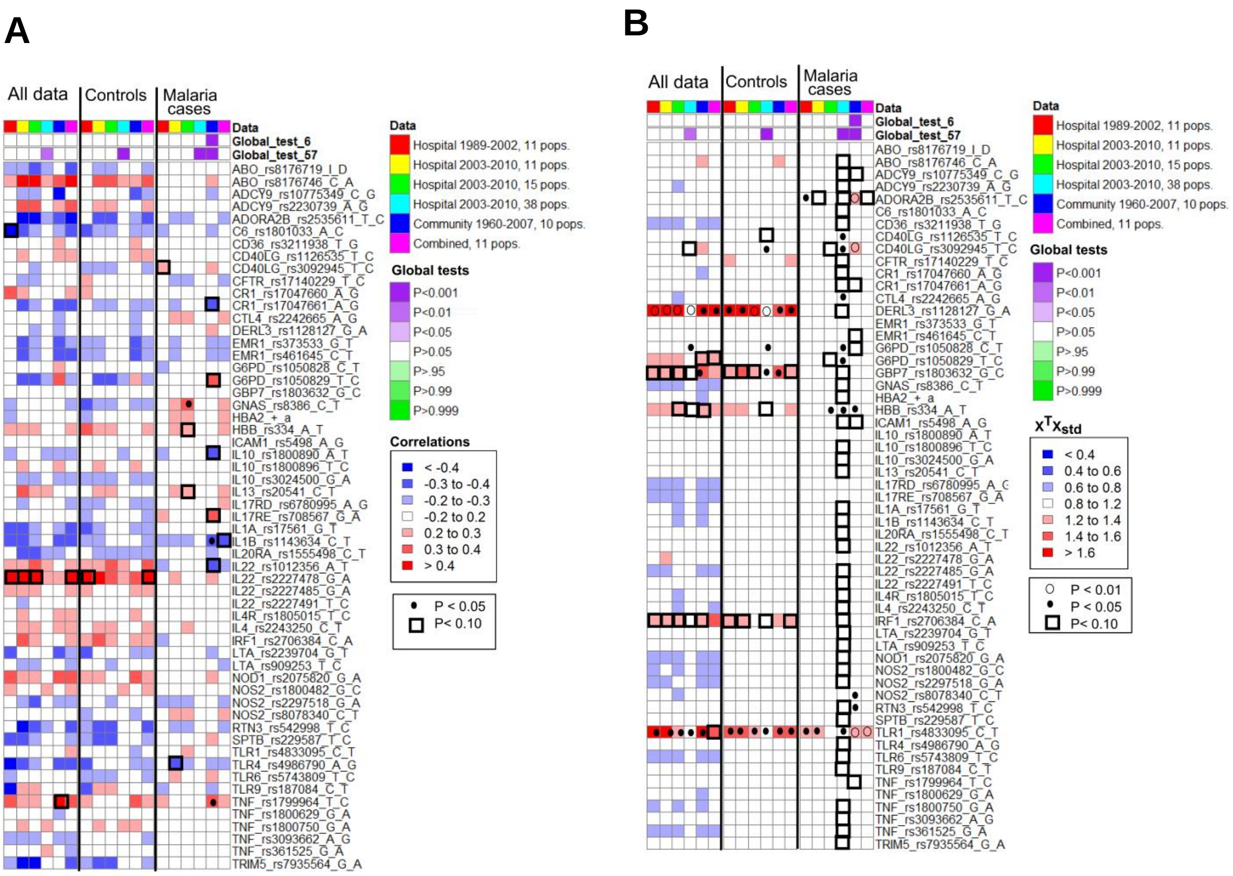

Fig. S4. Environmental correlations and population differentiation tests for candidate loci based on different genotype and malaria prevalence data sets. (*A*) Environmental correlations were computed for three genotype data sets (groups of columns separated by black vertical lines, labelled at top of figure) and six indices of malaria transmission intensity (top coloured row of the figure, top legend) for all 57 candidate loci (rows). Colouring of individual cells reflects strength of the correlation (red-blue legend). Where empirical *P*-values for individual SNPs were below 10% (two-tailed), cells are marked with black symbols (see lowest legend). Global tests of significance based on all 57 candidate loci, or on the top 6 candidate loci, are shown in the second and third rows of the figure with green and purple for global tests. Purple and green indicate, respectively, significantly lower and higher values for the global test statistic among candidate loci than random loci (purple-green legend). (*B*) As for (*A*) but for general population differentiation tests based on the X^TX statistic divided by its expected value (number of subpopulations) in order to standardise across data sets (X^TX_{std}). Since X^TX values for analysis using 38 subpopulations were generally below the theoretical expected value of 38 for random SNPs, but close to the theoretical expected value for most candidate loci, the colour of cells indicates non-departure from theoretical expected values even though many candidate SNPs showed significant X^TX statistics based on the empirical distribution of random loci.
