# Supplementary material for: Environmental Correlation Analysis for Genes Associated with Protection against Malaria
Source: Mol Biol Evol. 2016 Jan 6;33(5):1188–204. doi: 10.1093/molbev/msw004 (PMC4839215; doi:10.1093/molbev/msw004)
Supplement: Supplementary Data [file supp_msw004_suppl_data.zip › Supplementary Material - Tables Mackinnon MBE-15-1092 Dec 2015.pdf]

Supplementary Tables

For “Environmental correlation analysis for genes associated with protection against malaria”

Margaret J Mackinnon, Carolyne Ndila, Sophie Uyoga, Alex Macharia, Robert W. Snow, Gavin Band, Anna Rautanen, Kirk A. Rockett , Dominic P. Kwiatkowski and Thomas N. Williams, in collaboration with the MalariaGEN Consortium.

Table S1.

Table S1. Population allele frequencies, chromosomal locations and number of genotypes for the 57 SNPs in candidate malaria resistance genes.

Gene name	SNP	Chromosome	Position (bp)	Anc. allele[a]	Der. allele[a]	Local freq.[b]	Global freq,[c]
ABO	rs8176719	9	136132909	I	D	0.74	0.66
ABO	rs8176746	9	136131322	C	T	0.14	0.15
ADCY9	rs10775349	16	4079823	G	C	0.80	0.70
ADCY9	rs2230739	16	4033436	A	G	0.09	0.26
ADORA2B	rs2535611	17	15861332	T	C	0.11	0.04
C6	rs1801033	5	41199959	A	C	0.47	0.45
CD36	rs3211938	7	80300449	T	G	0.10	0.03
CD40LG	rs1126535	X	135730555	T	C	0.15	0.22
CD40LG	rs3092945	X	135729609	T	C	0.22	0.09
CFTR	rs17140229	7	117230283	T	C	0.35	0.11
CR1	rs17047660	1	207782856	A	G	0.16	0.08
CR1	rs17047661	1	207782889	A	G	0.67	0.20
CTL4	rs2242665	6	31839309	G	A	0.74	0.62
DERL3	rs1128127	22	24179132	G	A	0.49	0.26
EMR1	rs373533	19	6919624	G	T	0.47	0.27
EMR1	rs461645	19	6919753	T	C	0.53	0.72
G6PD	rs1050828	X	153764217	G	A	0.19	0.04
G6PD	rs1050829	X	153763492	T	C	0.41	0.09
GBP7	rs1803632	1	89582690	C	G	0.51	0.37
GNAS	rs8386	20	57485812	C	T	0.19	0.05
HBA	[d]	16	222846	αα	α–	0.40	-
HBB	rs334	11	5248232	A	T	0.08	0.03
ICAM1	rs5498	19	10395683	A	G	0.12	0.36
IL10	rs1800890	1	206949365	T	A	0.75	0.79
IL10	rs1800896	1	206946897	T	C	0.37	0.27
IL10	rs3024500	1	206940831	A	G	0.45	0.29
IL13	rs20541	5	131995964	G	A	0.22	0.27
IL17RD	rs6780995	3	57138419	G	A	0.60	0.53
IL17RE	rs708567	3	9960070	G	A	0.49	0.39
IL1A	rs17561	2	113537223	G	T	0.16	0.22
IL1B	rs1143634	2	113590390	C	T	0.12	0.13
IL20RA	rs1555498	6	137325847	C	T	0.46	0.85
IL22	rs1012356	12	68644618	A	T	0.50	0.49
IL22	rs2227478	12	68648622	G	A	0.61	0.66
IL22	rs2227485	12	68647713	G	A	0.42	0.48
IL22	rs2227491	12	68646521	A	G	0.60	0.55
IL4	rs2243250	5	132009154	C	T	0.79	0.47
IL4R	rs1805015	16	27374180	T	C	0.43	0.20
IRF1	rs2706384	5	131826880	C	A	0.38	0.57
LTA	rs2239704	6	31540141	G	T	0.13	0.35
LTA	rs909253	6	31540313	T	C	0.50	0.39

Table S1 cont.

NOD1	rs2075820	7	30492237	G	A	0.38	0.34
NOS2	rs1800482	17	26128509	G	C	0.92	0.98
NOS2	rs2297518	17	26096597	G	A	0.14	0.17
NOS2	rs8078340	17	26129212	C	T	0.21	0.13
RTN3	rs542998	11	63487386	T	C	0.42	0.74
SPTB	rs229587	14	65263300	T	C	0.33	0.53
TLR1	rs4833095	4	38799710	C	T	0.08	0.43
TLR4	rs4986790	9	120475302	A	G	0.04	0.06
TLR6	rs5743809	4	38830514	T	C	0.06	0.02
TLR9	rs187084	3	52261031	C	T	0.67	0.38
TNF	rs1799964	6	31542308	C	T	0.73	0.78
TNF	rs1800629	6	31543031	G	A	0.08	0.09
TNF	rs1800750	6	31542963	G	A	0.06	0.01
TNF	rs3093662	6	31544189	A	G	0.11	0.08
TNF	rs361525	6	31543101	G	A	0.07	0.06
TRIM5	rs7935564	11	5718517	G	A	0.44	0.46

Notes for table S1

a. Ancestral and derived alleles as reported in dbSNP on November 1, 2015. Environmental correlations and allele frequencies reported in this study relate to the derived allele.
b. Frequency of the derived allele in this study population.
c. Downloaded from dbSNP based on the 1000 Genomes Project.
d. Assayed by length polymorphisms rather than by SNPs (see Materials and Methods).

Table S2. Characteristics of genes showing the strongest environmental correlations with malaria for candidate malaria resistance loci and random SNP loci. Only the top 21 of the candidate loci are shown. For the non-random SNPs, 55 genes (including non-coding RNAs) that were found in the top 98 (1%) of random SNPs are shown.

SNP	Gene	[a]Anc. allele	[b]Der. allele	[c]Global freq.	[d]Local freq.	[e]ECA corr.	[f]ECA *P*-val.	[g]Corresp. with other malaria studies	[h]Associations with other traits	[i]Gene Description	[j]Protein function
**Candidate malaria resistance loci**											
rs2227478	*IL22*	C	T	0.66	0.61	0.43 (0.56)	0.06 (0.07)	- (7)[k]	Inflammation, autoimmunity, acute phase response	interleukin 22	Cytokine mediators of cellular inflammatory responses
rs8176746	*ABO*	C	T	0.15	0.14	0.33 (0.48)	0.16 (0.14)	- (12; 19; 16)	E-selectin levels, fibrin fragment D, cardiovascular disease, interleukin-6, metabolism, ICAM-1, depression	ABO blood group	Determines ABO blood group
rs2535611	*ADORA2B*	T	C	0.04	0.11	-0.31 (-0.35)	0.20 (0.29)		Asthma, cystic fibrosis, leukaemia, cholera	adenosine A2b receptor	Receptor that plays a role in relaxation of smooth muscle in the vasculature and intestines, and inhibits monocyte and macrophage function
rs4986790	*TLR4*	A	G	0.06	0.04	-0.29 (-0.46)	0.24 (0.15)	+(21)[l]	Atherosclerosis, asthma, inflammatory bowel disease, macular degeneration, liver disease	toll-like receptor 4	Pathogen recognition and activation of innate immune response
rs1801033	*C6*	A	C	0.45	0.47	-0.27 (-0.40)	0.28 (0.22)		Susceptibility to severe recurrent infections, e.g.,	complement component 6	Component of the complement cascade that helps lyse foreign

									Neisseria		cells
rs1143634	*IL1B*	C	T	0.13	0.12	-0.27 (-0.44)	0.29 ().18)		Many, including toxic shock syndrome, CNS leukaemia, klebsiella infection	interleukin 1, beta	Inflammatory cytokine
rs542998	*RTN3*	T	C	0.74	0.43	-0.26 (-0.24)	0.31 (0.48)	+ (18) - (20; 1)[m]	None	reticulon 3	May be involved in membrane trafficking and viral pathogenesis
rs1799964	*TNF*	C	T	0.78	0.73	-0.25 (-0.45)	0.32 (().16)	+ (11; 4)	Many, including rheumatoid arthritis,mCrohns disease, meliodisis, stomatitis	tumour necrosis factor	Proinflammatory cytokine
rs17561	*IL1A*	G	T	0.22	0.16	-0.25 (-0.44)	0.32 (0.18)	+ (9; 18)	Necrosis, tumours, inflammation, rheumatoid arthritis	interleukin 1, Alpha	Cytokine mediator of cellular inflammatory responses and haematopoiesis
rs7935564	*TRIM5*	G	A	0.46	0.44	-0.25 (-0.44)	0.33 (0.18)	- (18)	Immunodeficienc y, viral and retroviral infections	tripartite motif containing 5	Capsid-specific restriction factor that prevents infection from non-host-adapted retroviruses
rs8176719	*ABO*	I	D	?	0.74	-0.23 (-0.34)	0.36 (0.31)	All	E-selectin levels, fibrin fragment D, cardiovascular disease, interleukin-6, metabolism, ICAM-1, depression	ABO blood group	Determines ABO blood group
rs10775349	*ADCY9*	A	G	0.26	0.09	0.23 (0.31)	0.36 (0.35)	+(18; 1)[n]	Adenoma, cholera	adenylate cyclase 9	Formation of cAMP from ATP for signal transduction
rs17047661	*CR1*	A	G	0.20	0.67	-0.22	0.39	+ (10)	Lupus, malaria	complement	Glycoprotein on cell

						(-0.30)	(0.37)			components (3b/4b) receptor 1	surfaces, including erythrocytes that mediates binding to immune complexes
rs2075820	*NOD1*	G	A	0.34	0.38	0.21 (0.43)	0.40 (0.19)		Crohns disease, Blau syndrome, ulcerative colitis	nucleotide-binding oligomerization domain containing 1	Pattern recognition receptor in pathogen infection
rs461645	*EMR1*	T	C	0.72	0.53	0.21 (0.29)	0.42 (0.39)		None	adhesion G protein-coupled receptor E1	Signal transduction by adhesion molecules
rs373533	*EMR1*	G	T	0.27	0.47	-0.20 (-0.24)	0.44 (0.48)		None	adhesion G protein-coupled receptor E1	Signal transduction by adhesion molecules
rs229587	*SPTB*	A	G	0.53	0.33	-0.18 (-0.30)	0.41 (0.37)	+ (20)	Stroke, amyotrophic lateral sclerosis, iron	spectrin, beta, erythrocytic	Cell membrane organisation and stability
Rs1012356	*IL22*	A	T	0.44	0.50	0.19 (0.19)	0.46 (0.58)	- (7)[k]	Inflammation, autoimmunity, acute phase response	interleukin 22	Cytokine mediators of cellular inflammatory responses
rs334	*HBB*	A	T	0.03	0.08	0.19 (0.26)	0.40 (0.44)	All studies	Sickle cell disease	hemoglobin, Beta	Involved in oxygen transport from the lung to the various peripheral tissues
rs2706384	*IRF1*	G	T	0.57	0.38	0.18 (0.22)	0.47 (0.52)	+ (17; 16) - (8; 5)[o]	None	interferon regulatory factor 1	Transcriptional regulator affecting cellular responses
rs8386	*GNAS*	C	T	0.05	0.19	-0.15 (-0.26)	0.56 (0.44)	+ (1) - (10)	Bleeding, adrenal hyperplasia, colorectal cancer	GNAS complex locus	Modulators or transducers in various transmembrane signaling systems
**Random SNPs**											
rs4727622	*SRPK2*	A	C	0.28	0.23	-0.72	0.0000		None	SRSF protein kinase 2	Regulation of splicing
rs11875811	*MIR7153*	G	A	0.88	0.84	-0.68	0.0001		None	microRNA mir-7153	Post-transcriptional regulation of gene expression
rs4328444	*RBFOX1*	C	G	0.16	0.18	-0.67	0.0003		Spinocerebellar ataxia type 2	RNA binding protein, fox-1 homolog (C. elegans) 1	Alternative splicing

rs10504014	*IDO2*	C	T	0.58	0.69	0.67	0.0004		None	indoleamine 2,3-dioxygenase 2	Tryptothan metabolism, heme binding
rs9290240	*LOC105374191*	T	C	0.74	0.50	0.66	0.0005		None	Uncharacterised	Unknown
rs9867427	*LOC105376993*	A	G	0.22	0.15	-0.66	0.0006		None	Uncharacterised	Unknown
rs8048962	*CDH13*	T	C	0.43	0.43	0.64	0.0013	(3; 15)[p]	Lung cancer	cadherin 13	Calcium-dependent cell adhesion and signal transduction
rs7270295	*SLX4IP*	C	T	0.14	0.07	-0.63	0.0015		Acute lymphoblastic leukemia	SLX4 interacting protein	Unknown
rs1250203	*FN1*	A	C	0.17	0.45	0.63	0.0016		Glomerulopathy	fibronectin 1	Cell adhesion
rs545517	*CACNA2D3*	G	A	0.21	0.17	0.62	0.0021		Hereditary night blindness	calcium channel, voltage-dependent, alpha 2/delta subunit 3	Calcium channel activity
rs4508867	*PPARGC1A*	C	T	0.33	0.15	0.62	0.0022		Obesity, lipmatosis, breast cancer	peroxisome proliferator-activated receptor gamma, coactivator 1 alpha	Transcriptional regulator of energy metabolism
rs572481	*RBFOX1*	T	C	0.25	0.44	-0.62	0.0023		Spinocerebellar ataxia type 2	RNA binding protein, fox-1 homolog (C. elegans) 1	Alternative splicing
rs1355643	*POMGNT1*	T	C	0.04	0.13	0.62	0.0025		Muscular dystrophy	protein O-linked mannose N-acetylglucosaminyl-transferase 1 (beta 1,2-)	Glycosylation
rs10915816	*DNAH14*	T	C	0.29	0.59	0.62	0.0027		None	dynein, axonemal, heavy chain 14	Microtubule-associated motor protein complexes
rs17018509	*DTL*	G	T	0.04	0.10	0.61	0.0028		None	denticleless E3 ubiquitin protein ligase homolog (Drosophila)	Polyubiquitination
rs947360	*FAM155A*	T	G	0.26	0.49	0.61	0.0029		None	family with sequence similarity 155, member A	None

rs13358276	*CTNND2*	C	T	0.33	0.14	-0.61	0.0035		Prostate cancer, Alzheimer's disease, mental retardation	catenin (cadherin-associated protein), delta 2	Involved in cadherin-mediated cell adhesion
rs7761505	*PARK2*	G	A	0.39	0.53	0.61	0.0037		Parkinson disease	parkin RBR E3 ubiquitin protein ligase	Proteasomal degradation
rs1505595	*SYNPR*	G	A	0.28	0.40	-0.60	0.0040		None	synaptoporin	Possible channel protein of synaptic vesicles
rs35836	*PSMD6*	G	T	0.08	0.20	-0.60	0.0042		None	proteasome (prosome, macropain) 26S subunit, non-ATPase, 6	Proteasomal degradation
rs253	*LPL*	T	C	0.49	0.29	0.60	0.0043		Lipoprotein lipase deficiency, atherosclerosis, diabetes mellitus	lipoprotein lipase	Lipid metabolism and uptake
rs7032444	*GNA14*	T	G	0.23	0.08	0.60	0.0045		Pertussis	guanine nucleotide binding protein (G protein), alpha 14	Transmembrane signallling
rs7665508	*GABRB1*	A	G	0.38	0.29	-0.59	0.0047		None	gamma-aminobutyric acid (GABA) A receptor, beta 1	Chloride channel in nerve synapses
rs4678876	*STAC*	T	G	0.76	0.79	0.59	0.0048		None	SH3 and cysteine rich domain	Probably neuron signal transduction
rs12443587	*VAT1L*	C	T	0.45	0.14	-0.59	0.0051		None	vesicle amine transport 1-like	Unknown
rs1223629	*PLCE1*	C	T	0.17	0.44	0.59	0.0052		Early onset nephrotic syndrome and glomerulo-sclerosis	phospholipase C, epsilon 1	Hydrolysis of phospholipids into fatty acids
rs3133762	*C8orf37-AS1*	G	A	0.37	0.51	0.59	0.0053		None	Uncharacterised	Non-coding RNA
rs849584	*NRP2*	G	T	0.34	0.38	0.59	0.0054		Neuropathy	neuropilin 2	Receptor in vascular endothelium
rs6692452	*KLHL20*	G	A	0.89	0.55	0.59	0.0057		None	kelch-like family member 20	Adaptor for ubiquitination process and regulation,including

											epithelial cadherin signalling
rs3991132	*ZNF516*	C	T	0.03	0.06	-0.59	0.0059		None	zinc finger protein 516	Transcriptional regulator
rs11830407	*BICD1*	C	T	0.14	0.10	-0.59	0.0062		Lissencephaly	bicaudal D homolog 1 (Drosophila)	COPI-independent membrane transport from Golgi to ER
rs7940889	*DAGLA*	C	T	0.07	0.21	0.59	0.0063		Spinocerebellar ataxia	diacylglycerol lipase, alpha	Axonal growth
rs7542210	*LOC101929224*	T	C	0.75	0.83	-0.59	0.0064		None	None	Unknown
rs11084178	*ZNF611*	T	C	0.57	0.35	0.58	0.0066		None	zinc finger protein 611	Transcriptional regulator
rs4791574	*HS3ST3B1*	A	C	0.36	0.07	0.58	0.0069	(2)[p]	Periodontitis and palmoplantar keratoderma	heparan sulfate (glucosamine) 3-O-sulfotransferase 3B1	Modifies heparan sulphate, a binding receptor
rs11201865	*GRID1*	G	T	0.56	0.41	0.58	0.0070		None	glutamate receptor, ionotropic, delta 1	Glutamate transport in neuronal synapses
rs4745035	*TRPM3*	T	C	0.38	0.54	0.58	0.0073		Dentin sensitivity	transient receptor potential cation channel, subfamily M, member 3	Calcium signalling
rs209914	*COL9A2*	T	G	0.19	0.10	0.58	0.0075		Epiphyseal dysplasia	collagen, type IX, alpha 2	Collagen
rs10133089	*NRXN3*	C	T	0.01	0.07	-0.58	0.0078		Autism, alcohol dependence	neurexin 3	Receptor and cell adhesion
rs10267797	*LOC105375445*	C	T	0.55	0.29	-0.57	0.0080		None	None	Unknown
rs7732348	*LOC101929154*	C	T	0.56	0.68	-0.57	0.0081		None	Uncharacterized	Unknown
rs759438	*LOC101929231*	T	C	0.29	0.65	-0.57	0.0082		None	None	Unknown
rs7125475	*OPCML*	C	T	0.42	0.63	-0.57	0.0087		Ovarian cancer	opioid binding protein/cell adhesion molecule-like	Opioid receptor
rs13098678	*LOC105374250*	C	T	0.18	0.15	0.57	0.0088		None	None	None
rs12092071	*SSBP3*	T	C	0.73	0.58	0.57	0.0090		Thyroid lymphoma	single stranded DNA binding protein 3	DNA binding. Possible regulation of alpha 2(I) collagen gene
rs7845210	*CTHRC1*	A	C	0.22	0.49	0.57	0.0091		Chronic esophagitis	collagen triple helix repeat containing 1	Vascular remodelling upon injury

Table S2 cont.

rs6685721	*ADGRL2*	A	G	0.94	0.83	-0.57	0.0092	None	adhesion G protein-coupled receptor L2	Regulation of exocytosis
rs4530975	*LHFPL3*	C	T	0.17	0.17	0.57	0.0094	Smooth muscle tumour	lipoma HMGIC fusion partner-like 3	Transmembrane protein
rs7780969	*MAGI2*	T	C	0.91	0.76	0.56	0.0096	Ohtahara electroclinical syndrome	membrane associated guanylate kinase, WW and PDZ domain containing 2	Possible assembly of neurotransmitter receptors and cell adhesion proteins
rs11150285	*LOC102724084*	C	T	0.35	0.21	-0.56	0.0097	None	None	Unknown
rs1078368	*PDE4D*	G	A	0.49	0.49	0.56	0.0098	Acrodysostosis	phosphodiesterase 4D, cAMP-specific	Signal transduction
rs7144185	*RGS6*	C	T	0.50	0.17	-0.56	0.0100	Sick sinus syndrome	regulator of G-protein signaling 6	Signal transduction
rs10487939	*CNTNAP2*	G	A	0.04	0.07	0.56	0.0102	Tourette syndrome, schizophrenia, epilepsy, autism, ADHD	contactin associated protein-like 2	Cell adhesion and receptor in nerve system
rs11223225	*OPCML*	C	T	0.26	0.18	-0.56	0.0104	Ovarian cancer	opioid binding protein/cell adhesion molecule-like	Opioid receptor
rs884256	*RIMBP2*	C	T	0.79	0.89	-0.56	0.0105	CD40 ligand deficiency	RIMS binding protein 2	Synaptic transmission

Notes for table S2

a. Ancestral (Anc.) allele as recorded in the dbSNP database November 1, 2015 (6).
b. Derived (Der.) (i.e., non-ancestral) allele as recorded in the dbSNP database November 1, 2015 (6).
c. Frequency of the derived allele based on data from the 1000 Genomes Project, as recorded in dbSNP (6).
d. Frequency of the derived allele in the population studied here calculated on genotype data from control individuals only, i.e., the birth cohort.
e. Correlation (*r*) between malaria prevalence and allele frequency of the alternative allele in this study population as estimated by environmental correlation analysis (ECA) using the Bayenv2.0 package (14). Estimates are based on malaria prevalence calculated from all data and genotype data from all individuals (cases and controls). Values shown are for when data on the same number of genotyped individuals for candidate loci as for random loci (N = 5,214) were used. Values in parentheses below are for when genotypes from all control individuals were used (N = 10,957).

Table S2 cont.

f. Empirical *P*-values based on the distribution of *r* values for 9,756 random SNP loci calculated on genotype data from all individuals (*N* = 5,214). *P*-values based on the theoretical distribution for Pearson correlations are shown in parentheses below.
g. For candidate loci, this column reflects whether malaria case-control studies have reported a significant association with this SNP ($P <$ 0.05 for any mode of inheritance model). + or - indicate that the direction of association was, respectively, consistent or inconsistent with that from ECA. A blank indicates no significant associations were reported in all the journal articles found in PubMed that analysed this SNP and included the word 'malaria' in the Abstract. For random loci, this column records significant results from case-control studies, population tests or rodent malaria mouse genetic experiments.
h. Pertains to the whole gene (rather than the SNP) as recorded in the 'Disorders' channel in the GeneCards database (13).
i. Taken from GeneCards database (13).
j. Taken from GeneCards database (13).
k. The T allele is associated with high antibody levels: it is assumed here that this reflects higher susceptibility.
l. From a meta-analysis based on studies of many diseases, including three studies on malaria.
m. Alleles marked as major and minor in Table 2 of Touré et al. 2012 are incorrectly labelled in that paper.
n. The G allele is associated with hyperpyrexia: it is not clear whether hyperpyrexia reflects protection or susceptibility to malarial disease.
o. In Diakite et al. (2011), the derived allele was associated with inability to clear parasites.
p. The SNP analysed in this study was not included in these referenced studies and so concordance/discordance with the result here is unknown.

References for table S2

1. Apinjoh, TO, JK Anchang-Kimbi, C Njua-Yafi, AN Ngwai, RN Mugri, TG Clark, KA Rockett, DP Kwiatkowski, EA Achidi, GENC Malaria. 2014. Association of candidate gene polymorphisms and TGF-beta/IL-10 levels with malaria in three regions of Cameroon: a case-control study. *Malar J* 13:236.
2. Atkinson, A, S Garnier, S Afridi, F Fumoux, P Rihet. 2012. Genetic variations in genes involved in heparan sulphate biosynthesis are associated with Plasmodium falciparum parasitaemia: a familial study in Burkina Faso. *Malar J* 11:108.
3. Band, G, QS Le, L Jostins, M Pirinen, K Kivinen, M Jallow, F Sisay-Joof, K Bojang, M Pinder, G Sirugo, et al. 2013. Imputation-based meta-analysis of severe malaria in three African populations. *PLoS Genet* 9:e1003509.
4. Clark, TG, M Diakite, S Auburn, S Campino, AE Fry, A Green, A Richardson, K Small, YY Teo, J Wilson, et al. 2009. Tumor necrosis factor and lymphotoxin-alpha polymorphisms and severe malaria in African populations. *J Infect Dis* 199:569-575.
5. da Silva Santos, S, TG Clark, S Campino, MC Suarez-Mutis, KA Rockett, DP Kwiatkowski, O Fernandes. 2012. Investigation of host candidate malaria-associated risk/protective SNPs in a Brazilian Amazonian population. *PLoS One* 7:e36692.
6. dbSNP. www.ncbi.nlm.nih.gov/projects/SNP/

7. Dewasurendra, RL, P Suriyaphol, SD Fernando, R Carter, K Rockett, P Corran, D Kwiatkowski, ND Karunaweera, M Consortium. 2012. Genetic polymorphisms associated with anti-malarial antibody levels in a low and unstable malaria transmission area in southern Sri Lanka. *Malar J* 11:281.
8. Diakite, M, EA Achidi, O Achonduh, R Craik, AA Djimde, MS Evehe, A Green, C Hubbart, M Ibrahim, A Jeffreys, et al. 2011. Host candidate gene polymorphisms and clearance of drug-resistant Plasmodium falciparum parasites. *Malar J* 10:250.
9. Dunstan, SJ, KA Rockett, NT Quyen, YY Teo, CQ Thai, NT Hang, A Jeffreys, TG Clark, KS Small, CP Simmons, et al. 2012. Variation in human genes encoding adhesion and proinflammatory molecules are associated with severe malaria in the Vietnamese. *Genes Immun* 13:503-508.
10. Eid, NA, AA Hussein, AM Elzein, HS Mohamed, KA Rockett, DP Kwiatkowski, ME Ibrahim. 2010. Candidate malaria susceptibility/protective SNPs in hospital and population-based studies: the effect of sub-structuring. *Malar J* 9:119.
11. Flori, L, NF Delahaye, FA Iraqi, M Hernandez-Valladares, F Fumoux, P Rihet. 2005. TNF as a malaria candidate gene: polymorphism-screening and family-based association analysis of mild malaria attack and parasitemia in Burkina Faso. *Genes Immun* 6:472-480.
12. Fry, AE, MJ Griffiths, S Auburn, M Diakite, JT Forton, A Green, A Richardson, J Wilson, M Jallow, F Sisay-Joof, et al. 2008. Common variation in the ABO glycosyltransferase is associated with susceptibility to severe *Plasmodium falciparum* malaria. *Hum Mol Genet* 17:567-576.
13. Genecards. www.genecards.org
14. Gunther, T, G Coop. 2013. Robust identification of local adaptation from allele frequencies. *Genetics* 195:205-220.
15. Liu, X, Y Yunus, D Lu, F Aghakhanian, WY Saw, L Deng, M Ali, X Wang, FM Nor, TA Rahman, et al. 2015. Differential positive selection of malaria resistance genes in three indigenous populations of Peninsular Malaysia. *Hum Genet* 134:375-392.
16. MalariaGEN. 2014. Reappraisal of known malaria resistance loci in a large multicenter study. *Nat Genet* 46:1197-1204.
17. Mangano, VD, G Luoni, KA Rockett, BS Sirima, A Konate, J Forton, TG Clark, G Bancone, E Sadighi Akha, DP Kwiatkowski, et al. 2008. Interferon regulatory factor-1 polymorphisms are associated with the control of Plasmodium falciparum infection. *Genes Immun* 9:122-129.
18. Manjurano, A, TG Clark, B Nadjm, G Mtove, H Wangai, N Sepulveda, SG Campino, C Maxwell, R Olomi, KR Rockett, et al. 2012. Candidate human genetic polymorphisms and severe malaria in a Tanzanian population. *PLoS One* 7:e47463.
19. Panda, AK, SK Panda, AN Sahu, R Tripathy, B Ravindran, BK Das. 2011. Association of ABO blood group with severe falciparum malaria in adults: case control study and meta-analysis. *Malar J* 10:309.
20. Toure, O, S Konate, S Sissoko, A Niangaly, A Barry, AH Sall, E Diarra, B Poudiougou, N Sepulveda, S Campino, et al. 2012. Candidate polymorphisms and severe malaria in a Malian population. *PLoS One* 7:e43987.
21. Ziakas, PD, ML Prodromou, J El Khoury, E Zintzaras, E Mylonakis. 2013. The role of TLR4 896 A>G and 1196 C>T in susceptibility to infections: a review and meta-analysis of genetic association studies. *PLoS One* 8:e81047.

Table S3. Functional enrichment of genes among the top 10% of random SNPs for environmental correlations with malaria prevalence. Enrichment tests were performed using the overrepresentation test in Panther Version 10.0 (3) and EnrichNet (1) using different types of gene classification.

Method and classification type[a]	Pathway	Number in reference set[b]	Number in top 10%[c]	Expected number[d]	Over- or under-representation	Fold enrichment[d]	P-value[e]	Relevance[f]
**PANTHER Pathways[g]**								
	Ubiquitin proteasome pathway	6	3	0.78	+	3.84	0.045	i
	Cadherin signaling pathway	33	11	4.3	+	2.56	0.005	ii
**PANTHER GO-Slim Biological Process**								
	protein acetylation	6	3	0.78	+	3.84	0.045	iii
	cell-matrix adhesion	35	10	4.56	+	2.19	0.018	iv
	cell adhesion	158	31	20.58	+	1.51	0.016	
	cell-cell adhesion	113	22	14.72	+	1.49	0.042	
	biological adhesion	163	31	21.23	+	1.46	0.024	
	nervous system development	203	37	26.44	+	1.4	0.025	
	cellular protein modification process	240	43	31.26	+	1.38	0.021	
	system development	306	54	39.86	+	1.35	0.014	

Table S3 cont.

**PANTHER GO-Slim Molecular Function**								
	metallopeptidase activity	54	15	7.03	+	2.13	0.005	
	lipid transporter activity	37	10	4.82	+	2.07	0.025	v
	transporter activity	251	45	32.7	+	1.38	0.019	
	transmembrane transporter activity	230	40	29.96	+	1.34	0.039	
**PANTHER GO-Slim Cellular Component**								
	extracellular matrix	83	17	10.81	+	1.57	0.047	
**PANTHER Protein Class**								
	metalloprotease	53	15	6.9	+	2.17	0.005	
	protease	98	20	12.77	+	1.57	0.034	
	transporter	238	41	31	+	1.32	0.041	
**EnrichNet - Reactome**								
	NCAM1 interactions	43	6	NA	+	NA	0.017	vi
	axon guidance	160	13	NA	+	NA	0.001	vii
**EnrichNet - KEGG**								
	Arrhythmogenic right ventricular cardiomyopathy (ARVC)	73	8	NA	+	NA	0.011	viii
	Tight junction	130	10	NA	+	NA	0.014	ix

Table S3 cont.

	Adherens junction	72	6	NA	+	NA	0.109	x
	O-glycan biosynthesis	30	4	NA	+	NA	0.109	xi
**EnrichNet - InterPro**								
	IPR00998 MAM dom	15	4	NA	+	NA	0.031	xii
	IPR010909 PLAC dom (protease and lacunin)	19	5	NA	+	NA	0.011	xiii

Notes for table S3

a. For analyses by PANTHER (3), all the types of classification which were tested are shown in the table. For EnrichNet analyses (1), all available pathway classifications in the tool were used but only those with at least one pathway showing significance are reported in the table.
b. This column shows the number of genes in the genome in the pathway which were represented among the random loci surveyed by ECA in this study. A total of 2,802 protein-coding genes were represented among the 9,756 SNPs as well as the PANTHER database.
c. As above, but among the 365 genes represented by the top 976 (10%) SNPs by ECA. For EnrichNet analyses, only 307 of these genes mapped to the database.
d. This column shows the expected number of genes assuming no enrichment. This was not calculated for EnrichNet which ranks pathways according to levels of network connectivity.
e. For PANTHER, *P*-values were calculated by binomial test. For EnrichNet, *P*-values were calculated by Fisher's Exact test. Correction for multiple testing was not applied.
f. Relevance of the enriched pathway to the main findings of this study, namely, in cell-cell adhesion, extracellular signalling and glycosylated surface proteins, are given in the numbered footnotes. These were extracted from GeneCards (4). Gene sets without footnotes had substantial overlap with those with footnotes and so are covered by the latter.
g. These are mainly signalling pathways.

Notes on relevance (last column in table S3)

[i] Modification for protein degradation or signalling for receptor endocytosis. Genes in the top 10% were *UBE2V1* which plays a role in induction and expression of NFκB and MAPK-responsive inflammatory genes, *AIG1* which plays a role in amoebiasis and *PSMD6* which interacts with G-coupled protein receptor (GCPR) and B cell receptor signalling pathways.

[ii] Pathway that mediates cell-cell adhesion involving cadherins, catenins and cytoskeletal proteins such as actins. A key function of this pathway besides adhesion is extracellular signalling, usually by GCPRs. The 11 genes in this pathway in the top 10% by ECA encode the following cadherins: CDH5, expressed in the vascular endothelium and involved in cell-cell adhesion that maintains endothelial non-permeability, as well as signalling; CDH13, also expressed in the vasculature but which lacks the cytoplasmic and membrane domain and so is not involved in binding to the cell cytoskeleton, but appears to release signals that protect the vascular endothelium from apoptosis due to oxidative stress; three protocadherins (cadherin-related), FAT1, PCDH9 and PCDH15; three catenins, CTNND2 which is expressed in the brain and involved in NFκB pathway that regulates the inflammatory response, and CTNNA2 and CTNNA3 which probably bind actin but perhaps not cadherins; the extracellular signalling molecule WNT5B; the epidermal growth factor receptor ERBB4 which is a tyrosine kinase that is regulates gene expression, cytoskeletal rearrangement, anti-apoptosis and increased cell proliferation and is expressed at highest levels in brain, heart and kidney; and CSNK2A1, another kinase involved in the NFκB pathway and which regulates cell proliferation, cell differentiation and apoptosis, and for which there is evidence that its phosphorylation activity modifies the expression of malaria parasite proteins on the red cell surface and hence cytoadherence-related pathology (2).

[iii] The three genes in the top 10% were *DPP6* and *DPP10* which encode proteins that promote cell surface expression of the potassium channel KCND2; and DPF3, a transcription regulator that binds acetylated histones.

[iv] The ten genes in the top 10% include eight that code for proteins in the extracellular matrix, namely, two collagens (COL6A3 and COL6A5); FN1 which binds other extracellular matrix components including heparan sulfate and helps promote vasculature repair; LAMA2 and NTN1 (both linkers between matrix proteins); and THSD4, ADAMTSL3, PAPLN, all metallopeptidases with ADAM-like thrombospondin motifs that are involved in shedding of extracellular portions of transmembrane proteins in the extracellular matrix. The remaining two genes encode proteins for ANGPT4, a receptor for tyrosine-protein kinases that regulate endothelial cells in the vasculature in response to inflammation and structural damage; and KIRREL3, a cell junction protein expressed in brain and kidney that is involved in ultrafiltration.

[v] Contains genes coding for two neurexins, NRXN3 and CNTNAP2, which bind neurons together; two neuropilins, (NETO1 and NRP2) which are transmembrane glycoprotein receptors for semaphorins that regulate axon guidance and immune response, and also bind VEGF which is involved in angiogenesis; two cation channel ATPases (ATP11A, ATP8A2) coupled to the transport of aminophospholipids from the outer to the

inner leaflet of membranes; three complement component proteins thought to be transmembrane receptors and adhesion molecules or metallopeptidases (CSMD1, CSMD2, CSMD3), and an ABC transporter (ABCA2) probably involved in lipid transport that affects skin development. Thus this gene set is characterised by membrane transporters that appear to be involved in cell recognition and cell adhesion in the nervous system, with some involvement in angiogenesis.

[vi] The six genes among the top 10% in this set include those coding for neural cell adhesion molecule 1 (NCAM1), four collagens and the cell surface receptor GFRA1. NCAM1 is generally considered as a cell adhesion mediator, but also acts as a receptor for signal transduction.

[vii] The 13 genes in top the 10% encode a mixture of receptors for semaphorin proteins that regulate nervous system development, inflammation and cytoskeletal dynamics, and which bind extracellular matrix proteins such as collagen and laminin.

[viii] This phenotype results from a defect in cell-cell binding in the heart muscle. The gene set includes catenins and cadherins.

[ix] The tight junction complex adheres cells in the internal epithelium together through protein complexes that link the extracellular matrix to the cell cytoskeleton. The gene set includes encoders of cadherins, catenins, two protein phosphatases (PPP2R1A, PPP2R1B) and two protein C kinases (PRKCE, PRKCH) of the serine/threonine calcium-independent type which mediate cell adhesion to the extracellular matrix via integrin-dependent signalling involving angiotensin and which are linked to ischemia through interaction with occludin. The top 10% set of genes also encode proteins that include a myosin (MYH15) which binds actin, the junctional adhesion molecule JAM2, an adhesive ligand at the endothelial tight junctions which interacts with a variety of immune cell types, and CSNK2A1, a molecule which helps anchor parasite proteins into the erythrocyte membrane thereby mediating cytoadherence-related pathology (see relevance note ii). See reference (5) for a review of this junction's role in the permeability and inflammation of the vascular endothelium in relation to the blood brain barrier.

[x] As for the tight junction but also found in the endothelium. Included in the top 10% were genes coding for CTNNA2, CTNNA3 and CSNK2A1, as for the tight junction and a further three genes coding for SORBS1, a tyrosine phosphatase of the E3 ubiquitin ligase CBL which plays a role in insulin-stimulated glucose transport but which also may be involved in formation of actin stress fibers and focal adhesions; PTPRM, another tyrosine phosphatase involved in cell-cell aggregation and signalling; and IQGAP1, a GTPase activating protein involved in cell-cells signalling and actin dynamics.

[xi] O-linked glycosylation modifies proteins in the extracellular matrix that mediate cell-cell adhesion. The proteins encoded by this gene set include eleven N-acetyl-galactoseamine transferases of which four were found in the gene set for the top 10% of SNPs. The form of glycosylation mediated by this subset of transferases – the addition of galactoseamine – converts the H antigen in the ABO blood group system to the A antigen. (The addition of galactose converts it to the B antigen). In this study, we found increasing frequency of the B allele with malaria prevalence whereas case-control studies have generally found the B allele to render people more susceptible to malaria, especially

when coupled with the A allele in AB heterozygotes. However, the sub-family of glycosyltransferases responsible for the ABO glycosylation are not those in this gene set.

[xii] Occurs in several cell surface proteins and is likely to have an adhesive function.

[xiii] Occurs in many matrix proteins.

References for table S3

1. Glaab, E, A Baudot, N Krasnogor, R Schneider, A Valencia. 2012. EnrichNet: network-based gene set enrichment analysis. *Bioinformatics* 28:i451-i457.
2. Hora, R, DJ Bridges, A Craig, A Sharma. 2009. Erythrocytic casein kinase II regulates cytoadherence of Plasmodium falciparum-infected red blood cells. *J Biol Chem* 284:6260-6269.
3. Mi, H, A Muruganujan, JT Casagrande, PD Thomas. 2013. Large-scale gene function analysis with the PANTHER classification system. *Nat Protoc* 8:1551-1566.
4. Safran, M, I Dalah, J Alexander, N Rosen, T Iny Stein, M Shmoish, N Nativ, I Bahir, T Doniger, H Krug, et al. 2010. GeneCards Version 3: the human gene integrator.
5. Wallez, Y, P Huber. 2008. Endothelial adherens and tight junctions in vascular homeostasis, inflammation and angiogenesis. *Biochim Biophys Acta* 1778:794-809.
