# Supplementary material for: Environmental Correlation Analysis for Genes Associated with Protection against Malaria
Source: Mol Biol Evol. 2016 Jan 6;33(5):1188–204. doi: 10.1093/molbev/msw004 (PMC4839215; doi:10.1093/molbev/msw004)
Supplement: Supplementary Data [file supp_msw004_suppl_data.zip › Supplementary text 1 Mackinnon MBE-15-1092 Dec 2015.pdf]

Supplementary text 1. Accuracy of subpopulation estimates for allele frequency and malaria prevalence.

Allele frequencies

The median number of genotyped individuals per subpopulation for estimation of allele frequencies was 706 for the 57 candidate malaria resistance loci and 195 for random SNP loci (table 1 in main text) giving median standard errors of subpopulation alleles frequencies of 0.016 and 0.031, respectively.

Variance component analysis of allele frequencies found an average ratio of between to within subpopulation variance of 0.11: thus measurement error in allele frequencies dominated the total variance in allele frequencies in the denominator of the correlation coefficient, and hence the magnitude of environmental correlations.

The distributions of frequencies of the derived allele were not significantly different between candidate loci and random loci (*P* = 0.11 by two-sided Kolmogorov-Smirnov test, supplementary fig. S3*A*, Supplementary Material online). The relative numbers of genotyped individuals per subpopulation differed significantly between candidate and random loci (*P* = 0.04 by chi-squared test, χ^2 = 24.7 on 14 d.f., (supplementary fig. S3*B*, Supplementary Material online) but these differences were minor and are not expected to influence the results from ECA once the total number of genotyped individuals were equalized for the two sets of loci (see main text and supplementary text 2, Supplementary Material online).

Malaria prevalence

Median numbers of records per subpopulation for calculating malaria prevalence were 2,408, 897 and 683 for hospital admissions in 1989-2002, hospital admissions in 2003-2010 and community surveys in 1960-2007, respectively (table 1 in main text) giving median standard errors per subpopulation of 0.013, 0.016 and 0.022, respectively, and 0.012 when all malaria prevalence data were combined. Corresponding between-population standard deviations (i.e., measures of the total between-population variation) were 0.06, 0.09, 0.12 and 0.07. Thus, unlike allele frequencies, the between-population variance malaria prevalence in the denominator of *r* was not dominated by measurement error.
