# Supplementary material for: Environmental Correlation Analysis for Genes Associated with Protection against Malaria
Source: Mol Biol Evol. 2016 Jan 6;33(5):1188–204. doi: 10.1093/molbev/msw004 (PMC4839215; doi:10.1093/molbev/msw004)
Supplement: Supplementary Data [file supp_msw004_suppl_data.zip › Supplementary text 2 Mackinnon MBE-15-1092 Dec 2015.pdf]

Supplementary text 2. Effects of number of genotyped individuals on magnitude and accuracy of estimates of environmental correlations.

The influence of number of genotyped individuals (*N*) on estimates of environmental correlations (*r*) was examined by simulating data representative of the population structure, allele frequencies and observed environmental correlations for the 57 candidate loci in this study. To create a very large data set (termed here the 'infinite data set') from which subsamples with different values of *N* were drawn, the observed numbers of observations for each allele of the 57 candidate loci in each subpopulation in the genotyped control individuals (*N* = 10,597) were multiplied by 100. Augmenting the data in this way by the same amount for each subpopulation replicated the observed environmental correlations for the 57 loci in the infinite data set. These ranged from ~-0.7 to 0.7. Environmental correlation analysis was then performed on subsamples of size *N* drawn at random from the infinite data set for a range of values of *N* in 20 independent replicates. Drawing at random across the subpopulations in this way thus created sampling variation in subpopulation allele frequencies. The relationship between *N* and *r* was estimated by regressing the 57 values of *r* estimated from the subsample of genotypes, r_{sub}, on those from the infinite data set, $r_{full.}$ The slope of the regression line and the adjusted R-squared were used to describe, respectively, the effects of *N* on the magnitude and precision of estimates of *r.* In this simulation, environmental correlations were computed as the Pearson correlation of raw allele frequencies and malaria prevalence and thus ignored the genetic covariances among populations that are taken into account using the Bayenv method (see main text).

It was found that the magnitude of *r* scaled approximately linearly with *N* up to a value of N~15,000 after which the relationship became asymptotic (fig. 1*A*). Thus in this study, where N~5,300 genotypes were used for comparison of *r* values from candidate vs. random loci, estimates of *r* are expected to be approximately 0.5 of the real values. The precision of *r* estimates increased with *N* in a similar manner to that for *r* (fig. 1*B*).

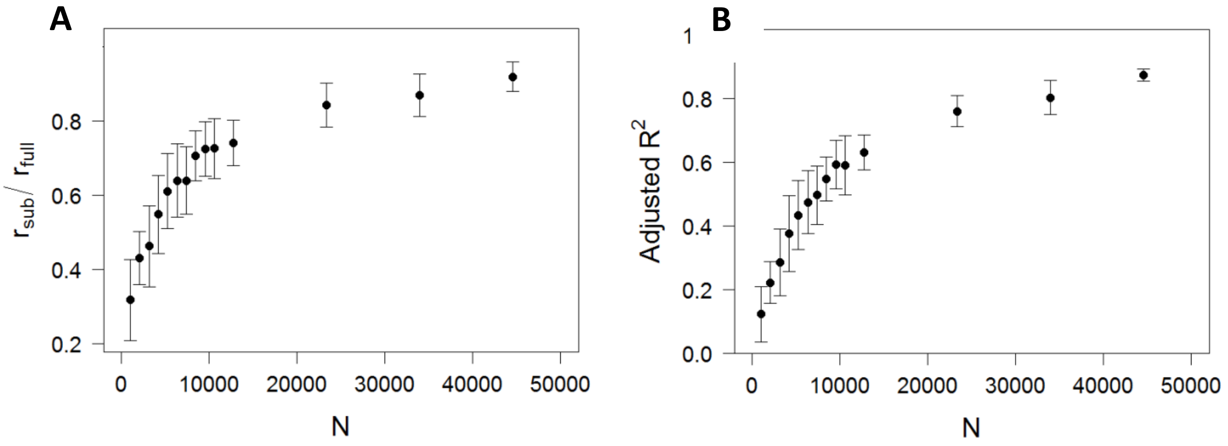

Fig. 1. Dependence of magnitude and precision of environmental correlation estimates on data set size. (*A*) The regression coefficient of estimates of *r* from subsets of data sized *N* (x-axis) relative to their true values (y-axis) as a function of *N. (B)* As for (*A*) but for precision of estimates of *r* as estimated by the adjusted R-squared statistic from linear regression of r_{sub} on *r.* Each point represents the mean over 20 replicate subsamples. Error bars extend one standard deviation either side of the mean.
